# Internet-Delivered Exposure and Response Prevention for Pediatric Tourette Syndrome: 12-Month Follow-Up of a Randomized Clinical Trial

**DOI:** 10.1001/jamanetworkopen.2024.8468

**Published:** 2024-05-03

**Authors:** Per Andrén, Filipa Sampaio, Helene Ringberg, Vera Wachtmeister, Moa Warnström, Kayoko Isomura, Kristina Aspvall, Fabian Lenhard, Charlotte L. Hall, E. Bethan Davies, Tara Murphy, Chris Hollis, Inna Feldman, Matteo Bottai, Eva Serlachius, Erik Andersson, Lorena Fernández de la Cruz, David Mataix-Cols

**Affiliations:** 1 Centre for Psychiatry Research, Department of Clinical Neuroscience, Karolinska Institutet, Stockholm, Sweden; 2 Stockholm Health Care Services, Region Stockholm, Stockholm, Sweden; 3 Department of Clinical Sciences, Lund, Lund University, Lund, Sweden; 4 Department of Public Health and Caring Sciences, Uppsala University, Uppsala, Sweden; 5 National Institute for Health and Care Research MindTech MedTech Co-Operative, Mental Health and Clinical Neurosciences, School of Medicine, University of Nottingham, Nottingham, United Kingdom; 6 National Institute for Health and Care Research Nottingham Biomedical Research Centre, Institute of Mental Health, Mental Health and Clinical Neurosciences, University of Nottingham, Nottingham, United Kingdom; 7 Great Ormond Street Institute of Child Health, University College London, London, United Kingdom; 8 Psychological and Mental Health Services, Great Ormond Street Hospital for Children, Great Ormond Street, London, United Kingdom; 9 Unit of Biostatistics, Institute of Environmental Medicine, Karolinska Institutet, Stockholm, Sweden

## Abstract

**Importance:**

Behavior therapy is a recommended intervention for Tourette syndrome (TS) and chronic tic disorder (CTD), but availability is limited and long-term effects are uncertain.

**Objective:**

To investigate the long-term efficacy and cost-effectiveness of therapist-supported, internet-delivered exposure and response prevention (ERP) vs psychoeducation for youths with TS or CTD.

**Design, Setting, And Participants:**

This 12-month controlled follow-up of a parallel group, superiority randomized clinical trial was conducted at a research clinic in Stockholm, Sweden, with nationwide recruitment. In total, 221 participants aged 9 to 17 years with TS or CTD were enrolled between April 26, 2019, and April 9, 2021, of whom 208 (94%) provided 12-month follow-up data. Final follow-up data were collected on June 29, 2022. Outcome assessors were masked to treatment allocation throughout the study.

**Interventions:**

A total of 111 participants were originally randomly allocated to 10 weeks of therapist-supported, internet-delivered ERP and 110 participants to therapist-supported, internet-delivered psychoeducation.

**Main Outcomes And Measures:**

The primary outcome was within-group change in tic severity, measured by the Total Tic Severity Score of the Yale Global Tic Severity Scale (YGTSS-TTSS), from the 3-month follow-up to the 12-month follow-up. Treatment response was defined as 1 (very much improved) or 2 (much improved) on the Clinical Global Impression–Improvement scale. Analyses were intention-to-treat and followed the plan prespecified in the published study protocol. A health economic evaluation was performed from 3 perspectives: health care organization (including direct costs for treatment provided in the study), health care sector (additionally including health care resource use outside of the study), and societal (additionally including costs beyond health care [eg, parent’s absenteeism from work]).

**Results:**

In total, 221 participants were recruited (mean [SD] age, 12.1 [2.3] years; 152 [69%] male). According to the YGTSS-TTSS, there were no statistically significant changes in tic severity from the 3-month to the 12-month follow-up in either group (ERP coefficient, −0.52 [95% CI, −1.26 to 0.21]; *P* = .16; psychoeducation coefficient, 0.00 [95% CI, −0.78 to 0.78]; *P* > .99). A secondary analysis including all assessment points (baseline to 12-month follow-up) showed no statistically significant between-group difference in tic severity from baseline to the 12-month follow-up (coefficient, −0.38 [95% CI, −1.11 to 0.35]; *P* = .30). Treatment response rates were similar in both groups (55% in ERP and 50% in psychoeducation; odds ratio, 1.25 [95% CI, 0.73-2.16]; *P* = .42) at the 12-month follow-up. The health economic evaluation showed that, from a health care sector perspective, ERP produced more quality-adjusted life years (0.01 [95% CI, −0.01 to 0.03]) and lower costs (adjusted mean difference −$84.48 [95% CI, −$440.20 to $977.60]) than psychoeducation at the 12-month follow-up. From the health care organization and societal perspectives, ERP produced more quality-adjusted life years at higher costs, with 65% to 78% probability of ERP being cost-effective compared with psychoeducation when using a willingness-to-pay threshold of US $79 000.

**Conclusions And Relevance:**

There were no statistically significant changes in tic severity from the 3-month through to the 12-month follow-up in either group. The ERP intervention was not superior to psychoeducation at any time point. While ERP was not superior to psychoeducation alone in reducing tic severity at the end of the follow-up period, ERP is recommended for clinical implementation due to its likely cost-effectiveness and support from previous literature.

**Trial Registration:**

ClinicalTrials.gov Identifier: NCT03916055

## Key Points

**Question:**

Is therapist-supported, internet-delivered exposure and response prevention (ERP) for youths with Tourette syndrome or chronic tic disorder efficacious and cost-effective in the long term, compared with therapist-supported, internet-delivered psychoeducation?

**Findings:**

In this follow-up study of a randomized clinical trial, 221 youths with Tourette syndrome or chronic tic disorder initially randomly allocated to therapist-supported, internet-delivered ERP or psychoeducation showed no tic severity change between the 3- and 12-month follow-up and no significant between-group differences. The use of ERP was cost-effective from a health care sector perspective (including health care resource use in and outside the study), but results were less conclusive from a societal perspective (additionally including costs beyond health care).

**Meaning:**

These results suggest that although ERP was not superior to psychoeducation alone in reducing tic severity at the end of the follow-up period, it is likely cost-effective.

## Teaser

This 12-month follow-up study of a randomized clinical trial assesses the efficacy and cost-effectiveness of therapist-supported, internet-delivered exposure and response prevention vs psychoeducation for youths with Tourette syndrome or chronic tic disorder.

## Introduction

Behavior therapy (BT) is a first-line intervention for Tourette syndrome (TS) and chronic tic disorder (CTD).^1,2^ There are 2 main BT protocols available, namely, the Comprehensive Behavioral Intervention for Tics and exposure and response prevention (ERP), of which the former has the strongest evidence base.^3,4,5^ Both treatments are typically delivered in person, and thus, their availability is limited.^6,7^ To make BT more accessible, several studies have investigated ways to deliver BT remotely.^8,9,10,11^ In a large UK randomized clinical trial (RCT)—the Online Remote Behavioural Intervention for Tics (ORBIT) trial^12^—224 young individuals with TS or CTD were randomized to 1 of two 10-week therapist-supported, internet-delivered interventions: ERP and psychoeducation. At the primary end point (posttreatment), internet-delivered ERP was superior to psychoeducation in reducing tic severity, requiring only minimal therapist resources.^13^ These results were maintained long-term (about 15 months after treatment).^14^ In a largely identical study involving 221 participants conducted in Sweden,^15^ internet-delivered ERP was not superior to psychoeducation in reducing tic severity at the primary end point (3 months posttreatment), but the ORBIT trial results were replicated on a secondary measure of treatment response.^16^ Overall, within-group results indicated clinically relevant improvements from both interventions, requiring minimal therapist time.

Long-term follow-up is particularly important in the evaluation of treatments for TS and CTD, as tics naturally wax and wane over time.^2^ Aside from the ORBIT trial,^14^ previous research has been restricted to a short follow-up duration (ie, up to 6 months after treatment)^4,5^ or following up only initial treatment responders.^3,4^ The present study reports on the prespecified^15^ 12-month follow-up of 221 participants in the Swedish RCT^16^ to establish the long-term efficacy and cost-effectiveness of internet-delivered ERP compared with psychoeducation.

## Methods

### Design

**Figure 1.**
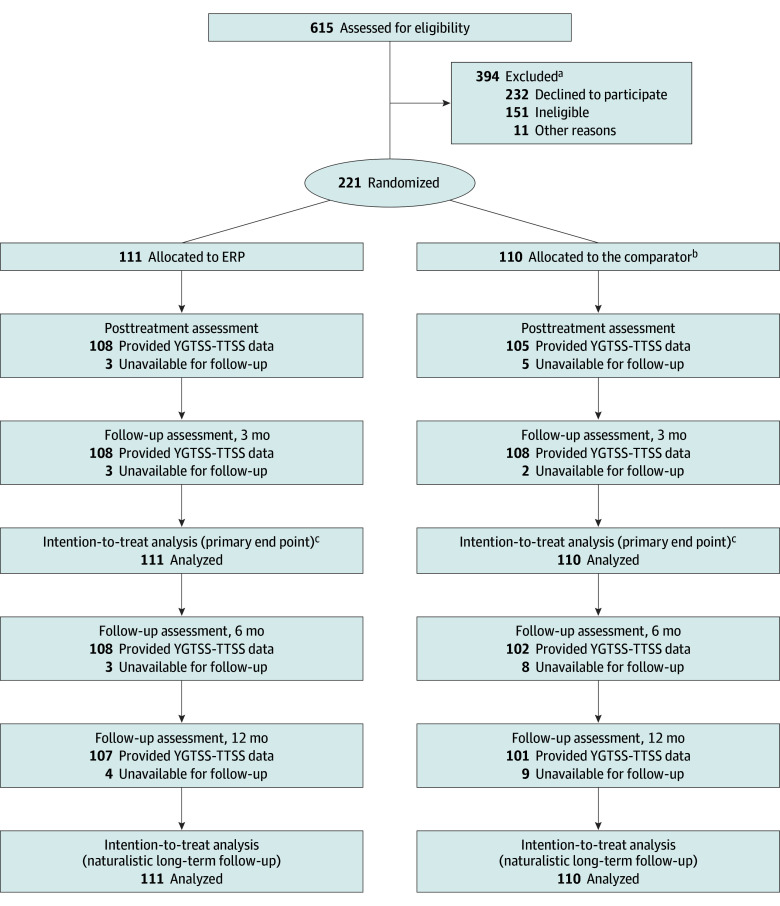
Participant Flow Diagram ^a^Further details on the reasons for exclusion are presented in the primary publication. ^b^The comparator is therapist-supported, internet-delivered psychoeducation. ^c^The analysis of the primary end point is presented in the primary publication. ERP indicates exposure and response prevention, defined as therapist-supported, internet-delivered exposure with response prevention for children and adolescents with Tourette syndrome or chronic tic disorder; YGTSS-TTSS, Yale Global Tic Severity Scale–Total Tic Severity Score.

This prespecified study reported controlled data from the 6-month and 12-month follow-up assessements of the participants in the original RCT (Figure 1). For further details on the study design, see the published study protocol,^15^ the primary publication,^16^ and the appended research protocol (Supplement 1). Ethical approval was obtained from the Swedish Ethical Review Authority. Written informed consent was collected from all participants and their legal guardians. Reporting follows the Consolidated Standards of Reporting Trials (CONSORT)^17^ and the Consolidated Health Economic Evaluation Reporting Standards (CHEERS) guidelines.^18^

### Participants and Randomization

Eligible participants were aged 9 to 17 years and had a *Diagnostic and Statistical Manual of Mental Disorders* (*Fifth Edition*) diagnosis of TS or CTD.^19^ Complete eligibility criteria are presented in eMethods 1 in Supplement 2. Participants were recruited across Sweden through clinician- and self-referrals. After assessments of tic severity and tic-related impairment (Yale Global Tic Severity Scale; YGTSS)^20^ and psychiatric comorbidities (Mini-International Neuropsychiatric Interview for children and adolescents),^21^ eligible participants were randomly assigned (1:1) to ERP or psychoeducation using randomly varying block sizes. Randomization was performed through an online service^22^ and monitored by an independent clinic trials unit.^23^

### Interventions

Both interventions were delivered during 10 weeks via an internet platform. Through separate logins, children and parents accessed intervention modules, including self-help texts, illustrations, videos, worksheets, and homework assignments. Therapist support was provided via asynchronous text messages inside the platform, supplemented with telephone calls when needed. Therapists were clinical psychologists or trainee psychologists trained in BT.

The ERP intervention was based on published treatment manuals.^24,25^ In ERP, participants initially practiced tic suppression (response prevention). Once they had gained mastery, they focused on the premonitory urges (ie, bothersome sensations preceding tic expression) to make the tic suppression more challenging (exposure and response prevention). The psychoeducation intervention was based on control interventions used in previous clinical trials of BT for TS and CTD.^3,4^ This comparator included psychoeducation (ie, about tic disorders and comorbid conditions) and behavioral exercises (eg, healthy habits and everyday routines). Details about both interventions are presented in Supplement 1. Families continued to have online access to all intervention modules (without therapist support) for the full 12-month follow-up period. After the 3-month follow-up, participants were free to pursue any treatment of their choice.

### Outcome Measures

The primary outcome measure was the YGTSS–Total Tic Severity Score (YGTSS-TTSS),^20^ a semistructured clinician-rated measure of tic severity (scores ranged from 0 to 50, with higher numbers indicating greater tic severity).^26^ All assessors were trained in the use of the YGTSS-TTSS (Supplement 1). Other clinician-rated measures included the YGTSS impairment score (scores ranged from 0 to 50 points, with higher scores indicating greater tic-related impairment),^20^ the Children’s Global Assessment Scale (CGAS; scores ranged from 1 to 100 points, with higher scores indicating higher functioning), ^27^ and the Clinical Global Impression Severity and Improvement scales (CGI-S/I; CGI-S scores ranged from 1 [no symptoms] to 7 [extreme symptoms]; CGI-I scores range from 1 [very much improved] to 7 [very much worse]).^28^ A score of 1 or 2 (much improved) on the CGI-I was used to define treatment response. Masking procedures are described in eMethods 2 in Supplement 2.

For the 6-month and 12-month follow-up assessements, clinician-rated measures were administered by assessors masked to group allocation via videoconference (385 [92%]), the telephone (29 [7%]) or face-to-face at the clinic (4 [1%]). Follow-up assessments were administered to both the child and at least 1 parent, or in some cases only the child (13 [3%]) or only a parent (54 [13%]). After each assessment, masked assessors recorded a guess of the participant’s group allocation.

Self- and parent-reported measures included the Parent Tic Questionnaire (PTQ; scores ranged from 0 to 224 points, with higher scores indicating greater tic severity),^29^ the Child and Adolescent Gilles de la Tourette Syndrome–Quality of Life scale (C&A-GTS-QOL; scores ranged from 0 to 135 points, with higher scores indicating lower quality of life),^30^ the Obsessive-Compulsive Inventory–Child Version (OCI-CV; scores ranged from 0 to 42 points, with higher scores indicating greater obsessive-compulsive severity),^31^ the Short Mood and Feelings Questionnaire child- and parent-reported versions (SMFQ-C and SMFQ-P, respectively; scores ranged from 0 to 26 points, with higher scores indicating greater depression),^32^ and the KIDSCREEN-10.^33^ Quality-adjusted life years (QALYs) were obtained through mapping KIDSCREEN-10^33^ data to the Child Health Utility 9 Dimensions (CHU9D) utility scores.^34^ Data on resource use were collected through the parent-reported Trimbos/iMTA (Institute for Medical Technology Assessment) questionnaire for costs associated with psychiatric illness (TiC-P).^35^

All self- and parent-reported outcome measures were completed through an online service. Data quality was monitored by an external clinical trials unit.^23^ A complete description of all outcome measures is available in Supplement 1.

### Health Economic Evaluation

A health economic evaluation was performed using 3 costing perspectives: (1) a health care organization perspective (including direct costs for treatment provided in the study, ie, therapist time), (2) a health care sector perspective (additionally including health care resource use outside the clinic and study, as well as medication costs), and (3) a societal perspective (additionally including costs beyond health care; eg, parents’ absenteeism from work). Two analyses were performed for each of the 3 perspectives: a cost-effectiveness analysis (using treatment response rate as the outcome) and a cost-utility analysis (using QALYs as the outcome).^36^ Incremental cost-effectiveness ratios (ICERs) operationalized as the cost per additional treatment responder or QALY were estimated. Further details on the health economic evaluation are presented in Supplement 1 and eMethods 3 in Supplement 2.

### Statistical Analysis

A power calculation was performed for the primary end point analysis of the original study (Supplement 1).^16^ Statistical analyses followed an a priori published statistical analysis plan (Supplement 1). Intention-to-treat, linear quantile mixed models were used to estimate median differences in the outcomes.^37,38,39^ First, within-group analyses evaluated whether the treatment effects at the 3-month follow-up were sustained at the 12-month follow-up. Second, between-group analyses investigated potential interaction effects of treatment and time from baseline to the 12-month follow-up. To enable comparisons with previous trials estimating mean differences, complementary intention-to-treat analyses using linear mixed models were performed. Quantile regression, logistic regression, and χ^2^ tests were used where appropriate. Effect sizes are presented as differences in median relative to the IQR (for median differences) and as Cohen *d* (for mean differences).^40^ Statistical significance was an α of .05 (2-sided). Analyses were performed using Stata, version 14.2 (StataCorp) and R, version 4.1.1 (R Project for Statistical Computing).

## Results

### Participants

Between April 26, 2019, and April 9, 2021, 221 participants were recruited, and 111 were randomly assigned to the ERP group and 110 to the comparator group (Figure 1). Final 12-month follow-up data were collected on June 29, 2022. The mean [SD] age of participants was 12.1 [2.3] years. Participants were predominantly boys (152 [69%]; 68 [31%] girls; 1 [0.4%] other gender) and fulfilled diagnostic criteria for TS (202 [91%]) or CTD (19 [9%]). The most common comorbid diagnoses were attention-deficit/hyperactivity disorder (34 [15%]) and anxiety disorders (31 [14%]). The participants were largely unmedicated at baseline (189 [86%]). Full participant characteristics are presented in eTable 1 in Supplement 2.

### Primary Outcome

Observed medians and means of the YGTSS-TTSS at each assessment point are presented in Table 1. Data loss on the YGTSS-TTSS was minimal (Table 1 and Figure 1).

**Table 1.** Observed Medians and Means at All Assessment Points for the Primary Outcome and a Selection of Secondary Outcomes

Outcome	No. of participants with available data	ERP (n = 111)^a^		Comparator (n = 110)^a^^,^^b^	
		Score, median (IQR)	Score, mean (SD)	Score, median (IQR)	Score, mean (SD)
**YGTSS-TTSS** ^c^					
Baseline	221	23 (18-26)	22.25 (5.60)	24 (19-27)	23.01 (5.92)
Posttreatment	213	19 (13-23)	18.53 (5.94)	20 (15-24)	19.27 (7.20)
3-mo Follow-up^d^	216	17 (11-21)	16.17 (6.82)	19 (12-23)	17.72 (7.11)
6-mo Follow-up	210	16 (10-21)	16.06 (6.98)	18 (11-23)	17.23 (8.18)
12-mo Follow-up	208	15 (9-21)	14.93 (7.70)	17 (11-23)	16.73 (8.30)
**YGTSS impairment score** ^e^					
Baseline	221	20 (10-20)	18.38 (7.08)	20 (10-20)	18.73 (7.79)
Posttreatment	213	10 (0-20)	10.65 (8.68)	10 (0-20)	11.52 (9.59)
3-mo Follow-up^d^	216	10 (0-10)	7.68 (8.82)	10 (0-10)	8.70 (8.10)
6-mo Follow-up	210	10 (0-10)	6.85 (7.81)	10 (0-10)	7.84 (8.97)
12-mo Follow-up	208	0 (0-10)	6.54 (8.14)	0 (0-10)	6.14 (8.12)
**CGI-S score** ^f^					
Baseline	221	4 (4-5)	4.08 (0.74)	4 (4-5)	4.19 (0.72)
Posttreatment	213	4 (3-4)	3.50 (0.86)	4 (3-4)	3.69 (0.91)
3-mo Follow-up^d^	216	3 (3-4)	3.24 (0.92)	4 (3-4)	3.49 (0.90)
6-mo Follow-up	210	3 (2-4)	3.10 (0.93)	3 (3-4)	3.32 (1.11)
12-mo Follow-up	208	3 (2-4)	2.97 (0.96)	3 (3-4)	3.25 (1.13)
**PTQ score** ^g^					
Baseline	221	32 (19-44)	34.33 (19.06)	34 (21-51)	38.04 (23.27)
Midtreatment^h^	210	22 (13-39)	25.73 (16.14)	26 (15-41)	29.83 (18.82)
Posttreatment	214	17 (10-30)	21.08 (15.75)	19.5 (11-36.5)	25.05 (18.18)
3-mo Follow-up^d^	211	14 (6-25)	19.84 (17.92)	19 (7.5-37.5)	23.51 (18.14)
6-mo Follow-up	206	14 (6-25)	18.17 (16.18)	17 (8-37)	24.18 (20.08)
12-mo Follow-up	203	11 (6-22)	16.76 (15.97)	16 (9-27)	20.76 (17.04)
**C&A-GTS-QOL score** ^i^					
Baseline	221	27 (17-39)	29.11 (15.06)	27.5 (18-43)	30.54 (16.54)
Posttreatment	212	15 (8-28.5)	19.68 (15.48)	20.5 (12-31)	22.86 (15.71)
3-mo Follow-up^d^	208	16 (8-28)	19.76 (16.26)	17 (9-27)	20.05 (15.72)
6-mo Follow-up	195	14 (7-26)	18.31 (15.20)	17 (9-30)	21.22 (16.69)
12-mo Follow-up	194	17 (9-27)	20.69 (17.54)	16 (9-26)	19.98 (15.19)

Abbreviations: C&A-GTS-QOL, Child and Adolescent Gilles de la Tourette Syndrome–Quality of Life scale; CGI-S, Clinical Global Impression-Severity; ERP, exposure and response prevention; PTQ, Parent Tic Questionnaire; TTSS, Total Tic Severity Score; YGTSS, Yale Global Tic Severity Scale.

^a^ Observed values calculated from completer data.

^b^ Defined as therapist-supported, internet-delivered psychoeducation.

^c^ Scores range from 0 to 50 points, with higher numbers indicating greater tic severity.

^d^ Primary end point.

^e^ Scores range from 0 to 50 points, with higher scores indicating greater tic-related impairment.

^f^ Scores range from 1 (no symptoms) to 7 (extreme symptoms).

^g^ Scores range from 0 to 224 points, with higher scores indicating greater tic severity.

^h^ Midtreatment indicates 5 weeks into the treatment.

^i^ Scores range from 0 to 135 points, with higher scores indicating lower quality of life.

During the follow-up phase, tic severity as measured by the YGTSS-TTSS was reduced a mean of 1.24 raw points in the ERP group and 0.99 raw points in the comparator (Table 1). These reductions were not statistically significant in within-group linear quantile mixed model analyses (ERP coefficient, −0.52 [95% CI, −1.26 to 0.21]; *P* = .16; comparator coefficient, 0.00 [95% CI, −0.78 to 0.78]; *P* > .99) (Table 2), indicating no change compared with the acute phase of the study for both groups. Bootstrapped within-group effect sizes (medians relative to the IQR) were 0.10 (95% CI, −0.05 to 0.24) for ERP and 0.00 (95% CI, −0.14 to 0.14) for the comparator. By contrast, the prespecified complementary linear mixed model found a significant improvement in the YGTSS-TTSS score during the follow-up phase in the ERP group (coefficient, −0.64 [95% CI, −1.21 to −0.07]; *P* = .03) (eTable 2 in Supplement 2), but no statistically significant improvement for the comparator (eTable 2 in Supplement 2).

**Table 2.** Results of Linear Quantile Mixed Models for the Primary Outcome and a Selection of Secondary Outcomes

Outcome	Intention-to-treat linear quantile mixed models								
	Within-group analysis ERP			Within-group analysis comparator^a^			Interaction between treatment and time		
	Coefficient (95% CI)	*P* value	Effect size (95% CI)^b^	Coefficient (95% CI)	*P* value	Effect size (95% CI)^b^	Coefficient (95% CI)	*P* value	Effect size (95% CI)^b^
**YGTSS-TTSS**									
Baseline to 12-mo follow-up	−1.46 (−1.88 to −1.05)	<.001^c^	0.49 (0.34 to 0.64)	−1.49 (−2.08 to −0.91)	<.001^c^	0.50 (0.33 to 0.67)	−0.38 (−1.11 to 0.35)	.30	0.13 (−0.12 to 0.37)
3-mo to 12-mo Follow-up	−0.52 (−1.26 to 0.21)	.16	0.10 (−0.05 to 0.24)	0.00 (−0.78 to 0.78)	>.99	0.00 (−0.14 to 0.14)	−0.55 (−1.33 to 0.24)	.17	0.10 (−0.08 to 0.28)
**YGTSS impairment**									
Baseline to 12-mo follow-up	−2.95 (−3.98 to −1.93)	<.001^c^	0.59 (0.32 to 0.86)	−3.17 (−4.02 to −2.31)	<.001^c^	0.63 (0.41 to 0.86)	0.16 (−0.55 to 0.86)	.67	0.03 (−0.14 to 0.20)
3-mo to 12-mo Follow-up	0.00 (−1.05 to 1.05)	>.99	0.00 (−0.42 to 0.42)	−0.13 (−0.48 to −0.21)	.45	0.27 (−0.65 to 0.70)	0.66 (−0.76 to 2.08)	.36	−0.13 (−0.49 to 0.23)
**CGI-S **									
Baseline to 12-mo follow-up	−0.27 (−0.42 to −0.12)	<.001^c^	1.08 (0.25 to 1.90)	−0.17 (−0.36 to 0.02)	.08	0.68 (−0.11 to 1.47)	0.03 (−0.16 to 0.21)	.28	−0.11 (−0.80 to 0.58)
3-mo to 12-mo Follow-up	0.00 (0.00 to 0.00)	>.99	0.00 (−0.03 to 0.03)	−0.01 (−0.07 to 0.05)	.78	0.02 (−0.11 to 0.15)	0.14 (0.06 to 0.21)	.00 ^c^	−0.27 (−0.55 to 0.01)
**PTQ**									
Baseline to 12-mo follow-up	−2.68 (−3.49 to −1.88)	<.001^c^	0.52 (0.39 to 0.65)	−2.45 (−3.26 to −1.64)	<.001^c^	0.47 (0.28 to 0.66)	−0.10 (−1.63 to 1.44)	.90	0.02 (−0.22 to 0.26)
3-mo to 12-mo Follow-up	−1.03 (−2.35 to 0.29)	.13	0.09 (−0.04 to 0.22)	−0.67 (−2.31 to 0.97)	.42	0.06 (−0.11 to 0.23)	0.22 (−2.50 to 2.95)	.87	−0.02 (−0.22 to 0.19)
**C&A-GTS-QOL**									
Baseline to 12-mo follow-up	−1.68 (−2.37 to −0.99)	<.001^c^	0.16 (0.09 to 0.23)	−1.50 (−2.16 to −0.85)	<.001^c^	0.29 (0.11 to 0.46)	0.18 (−0.98 to 1.33)	.77	−0.04 (−0.31 to 0.23)
3-mo to 12-mo Follow-up	0.56 (−0.99 to 2.10)	.48	−0.06 (−0.21 to 0.10)	0.73 (−0.71 to 2.17)	.32	−0.08 (−0.23 to 0.08)	0.06 (−1.79 to 1.90)	.95	−0.01 (−0.23 to 0.22)

Abbreviations: C&A-GTS-QOL, Child and Adolescent Gilles de la Tourette Syndrome–Quality of Life scale; CGI-S, Clinical Global Impression–Severity scale; ERP, exposure and response prevention; PTQ, Parent Tic Questionnaire; TTSS, Total Tic Severity Score; YGTSS, Yale Global Tic Severity Scale.

^a^ Defined as therapist-supported, internet-delivered psychoeducation.

^b^ Bootstrapped effect sizes, interpreted as differences in median relative to the IQR, are derived from the linear quantile mixed models.

^c^ Significant at an α level of .05.

**Figure 2.**
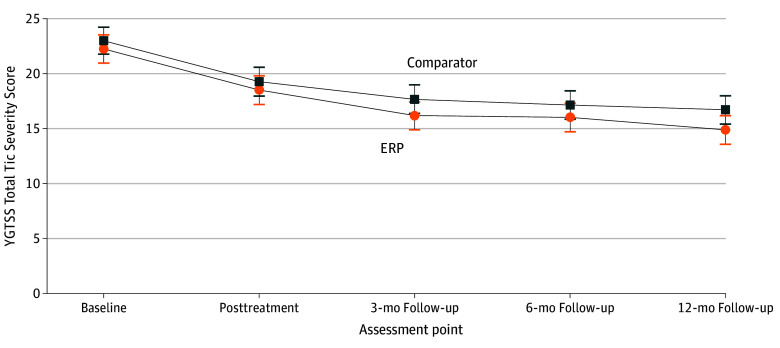
Estimated Mean Scores on the Yale Global Tic Severity Scale–Total Tic Severity Score (YGTSS-TTSS) From a Linear Mixed Model Including All 5 Assessment Points Follow-up assessements were 3, 6, and 12 months after treatment. ERP indicates exposure and response prevention; comparator, therapist-supported, internet-delivered psychoeducation. Error bars represent 95% CIs.

From baseline to the 12-month follow-up, tic severity as measured by the YGTSS-TTSS was reduced a mean of 7.32 raw points in the ERP group and 6.28 raw points in the comparator (both statistically significant reductions in within-group analyses; Table 1 and Table 2). A between-group linear quantile mixed model found no significant interaction effect between treatment and time on the YGTSS-TTSS between the same assessment points (coefficient, −0.38 [95% CI, −1.11 to 0.35]; *P* = .30) (Table 2). Similarly, the prespecified complementary linear mixed model (coefficient, −0.24 [95% CI, −0.61 to 0.13]; *P* = .21) was not statistically significant (eTable 2 in Supplement 2). Figure 2 depicts the estimated means at all assessment points.

### Secondary Outcomes

At the 6-month follow-up, we observed a statistically significant between-group difference (odds ratio, 1.96 [95% CI, 1.13-3.41]; *P* = .02) in the percentage of treatment responders (ERP, 57 responders [53%]; comparator, 37 responders [36%]). This difference was not statistically significant at the 12-month follow-up (ERP, 59 responders [55%]; comparator, 50 responders [50%]; odds ratio, 1.25 [95% CI, 0.73-2.16]; *P* = .42).

Observed medians and means of secondary outcomes at each assessment point are presented in Table 1 and eTable 3 in Supplement 2. Within-group linear quantile mixed model analyses showed that previously reported improvements^16^ on the YGTSS impairment score, the PTQ, the C&A-GTS-QOL, the CGAS, the OCI-CV, the SMFQ-C, and the SMFQ-P at the 3-month follow-up were unchanged up to the 12-month follow-up for both groups (Table 2; eTable 4 in Supplement 2). Similarly, the previously reported improvements on the CGI-S and KIDSCREEN-10 in the ERP group only were also unchanged up to the 12-month follow-up (Table 2; eTable 4 in Supplement 2).

Between-group linear quantile mixed model analyses using data from all 5 assessment points identified no significant interaction effects between treatment and time on any outcome measure (Table 2; eTable 4 in Supplement 2). Results of all complementary linear mixed model analyses are shown in eTable 2 in Supplement 2.

### Sensitivity Analyses and Masking Integrity

Between the 3-month and 12-month follow-up assessements, 27 participants (12%) either received face-to-face BT (ERP, habit reversal training, or a combination of both) or altered their TS or CTD medication (started, changed dosage, or stopped treatment). Four participants in the ERP group (4%) received a mean (SD) of 8.00 (3.56) sessions, while 12 participants in the comparator group (11%) received a mean (SD) of 7.58 (4.19) sessions. Further, in the ERP group, 5 participants (5%) started TS or CTD medication treatment or increased dosage, 1 participant (1%) decreased dosage, and 2 participants (2%) made several medication changes. In the comparator group, 1 participant (1%) started TS or CTD medication treatment and 2 participants (2%) made several medication changes.

Linear quantile mixed model sensitivity analyses excluding 27 participants with treatment changes during the follow-up showed results similar to the total sample, both in within-group analyses (ERP coefficient, −0.47 [95% CI, −1.19 to 0.26]; *P* = .20 vs comparator coefficient, 0.00 [95% CI, −0.97 to 0.97]; *P* = >.99; from the 3-month to the 12-month follow-up) and in an interaction analysis of treatment and time (coefficient, 0.00 [95% CI, −0.61 to 0.61]; *P* = .99; from baseline to the 12-month follow-up).

At both follow-up assessements, assessors’ guess of group allocation was no better than chance (6-month follow-up: 55%; χ^2^, 1.92; *P* = .17 and 12-month follow-up: 56%; χ^2^, 3.25; *P* = .07) (eTable 5 in Supplement 2).

### Post Hoc Analyses

To examine the impact of age on treatment outcomes, the total sample was split into 2 groups by median age (9-11 years for 124 participants, and 12-17 years for 97 participants). Tic severity improvements (YGTSS-TTSS) were similar in both age groups, with no differences between the intervention groups (9-11 years, −0.40 [95% CI, −1.49 to 0.71]; *P* = .48 and 12-17 years, −0.32 [95% CI, −1.04 to 0.40]; *P* = .38). In an additional post hoc analysis stratified by responder status at the primary end point (both groups combined), 82 treatment responders showed no change in tic severity (YGTSS-TTSS, 0.32 [95% CI, −0.40 to 1.03]; *P* = .38), whereas 139 nonresponders improved during the follow-up period [YGTSS-TTSS, −1.10 [95% CI, −1.59 to −0.61]; *P* = <.001).

### Health Economic Evaluation

Following the primary publication of the trial results,^16^ to avoid skewed cost estimates, the health economic evaluation excluded 1 participant in the comparator group due to a serious treatment-unrelated adverse event (meningitis). Baseline KIDSCREEN-10 scores, CHU9D utility scores, total health care costs, and total societal costs are presented in eTable 6 in Supplement 2. Unit costs are presented in eTable 7 in Supplement 2. Total discounted mean costs during the trial period (from baseline to the 12-month follow-up) and mean differences between the ERP and comparator groups are presented in eTable 8 in Supplement 2. From the health care organization perspective, since no further trial interventions were provided during the follow-up period, the intervention costs at the 12-month follow-up were identical to those previously reported,^16^ with significantly higher costs for the ERP group (US $117.38) than for the comparator group (US $102.23; mean difference $15.14 [95% CI, $5.08-$25.20]) (eTable 8 in Supplement 2). From the health care sector perspective, ERP cost $84.48 less per child than the comparator, although this was not statistically significant (adjusted mean difference −$84.48 [95% CI, −$440.20 to $977.60]) (eTable 8 in Supplement 2). From the societal perspective, ERP equated to an additional expense of $127.66 per child, although this was not statistically significant (adjusted mean difference $127.66 [95% CI, −1061.62 to 2562.26]) (eTable 8 in Supplement 2). CHU9D utility scores per assessment point and total QALYs are presented in eTable 9 in Supplement 2.

**Figure 3.**
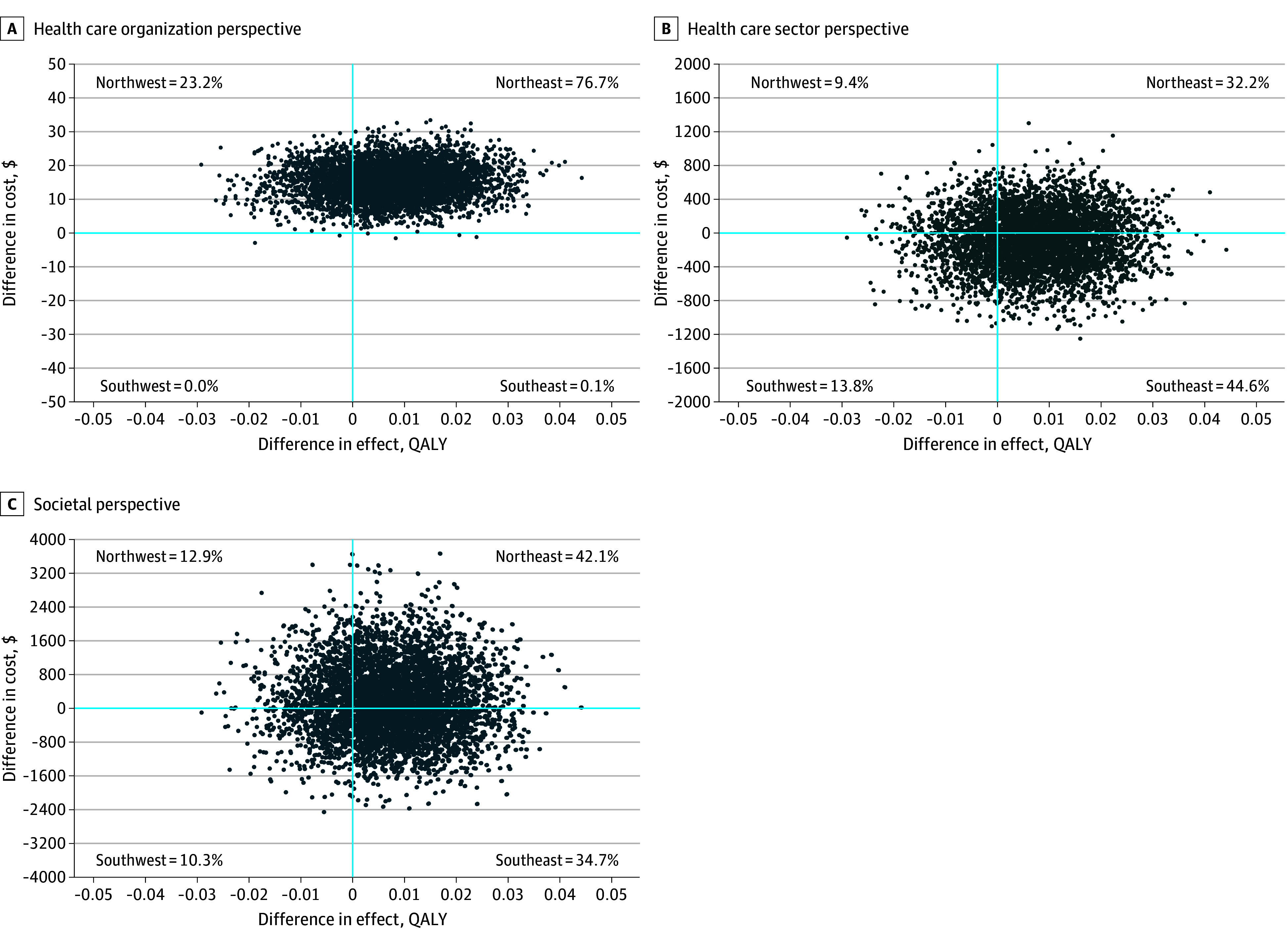
Cost-Effectiveness Planes With Quality-Adjusted Life Years (QALYs) as the Outcome for 3 Costing Perspectives All 3 cost-effectiveness planes compare exposure and response prevention (ERP) to the comparator of therapist-supported, internet-delivered psychoeducation, using QALYs as the outcome. A, Includes costs of the ERP or comparator interventions (ie, the therapist-support time). B, Includes costs of the ERP or comparator interventions, health care visits, and medication or supplements. C, Includes costs of the ERP or comparator interventions, health care visits, medication or supplements, and other sector costs (eg, productivity losses or child school absenteeism). The probability of the ERP group showing QALY gains at higher costs is in the northeast quadrant; the equivalent probability at lower costs is in the southeast quadrant. The probability of the comparator showing QALY gains at higher costs is in the northwest quadrant; the equivalent probability at lower costs is in the southwest quadrant.

The cost-utility analysis at the 12-month follow-up showed small nonsignificant gains in QALYs for the ERP group (0.01 [95% CI, −0.01 to 0.03]) at lower (health care sector perspective) or higher (health care organization and societal perspectives) costs (Figure 3; eTable 10 in Supplement 2). The cost-effectiveness analysis showed nonsignificantly higher treatment response rates for the ERP group (0.05 [95% CI, −0.08 to 0.19]) at lower or higher costs, depending on the costing perspective (eTable 10 and eFigure 1 in Supplement 2). The use of ERP dominated the comparator from the health care sector perspective, producing more QALYs and higher treatment response rates at a lower cost. The ICER estimates varied between $295 for the health care organization perspective and $2484 for the societal perspective per additional treatment responder and between $2150 for the health care organization perspective and $18 123 for the societal perspective per QALY gained (eTable 10 in Supplement 2). The latter interval of ICERs was below a willingness-to-pay threshold of $79 000 for 1 gained QALY,^41^ at which threshold ERP showed a 65% to 78% probability of being cost-effective (eFigure 2 and eFigure 3 in Supplement 2 show cost-effectiveness acceptability curves).

## Discussion

In this controlled follow-up study of 221 children and adolescents with TS or CTD participating in an RCT comparing internet-delivered ERP with psychoeducation, there were no statistically significant changes in tic severity from the 3-month to the 12-month follow-up in either group. At the 12-month follow-up, ERP was not superior to psychoeducation in reducing tic severity, and the percentage of treatment responders was similar. Responder rates increased from 47% (3-month follow-up) to 55% (12-month follow-up) in the ERP group and more notably, from 29% to 50% in the psychoeducation group, with no between-group differences 12 months after treatment.

The raw tic severity improvement on the YGTSS-TTSS from baseline to the 12-month follow-up was 7.32 points for the ERP group and 6.28 points for psychoeducation. The sister ORBIT trial presented data up to 18 months after randomization (equivalent to 15 months after treatment) and showed a similar tic severity improvement for the ERP group to that in the present study (6.9 raw points on the YGTSS-TTSS) but a slightly smaller improvement for psychoeducation (4.5 points).^13,14^ A similar pattern was shown for the treatment response rates, with both studies classifying 55% of participants as treatment responders in the ERP group, while there was a difference for psychoeducation (50% in the current study, 41% in ORBIT). The somewhat unexpected larger effect of the comparator in our study may be due to a range of reasons, such as a sample population with milder symptom severity and the use of more experienced therapists. Considering that symptoms improved similarly in both groups, the study design cannot rule out that factors other than the provided interventions contributed to the measured effects. Natural improvement over time could be one such explanation, which may be particularly likely in the long-term, given that tics generally decrease with age.^42^ Such an effect could have contributed to eroding potential differences between the groups, especially for a sample population with relatively mild symptom severity and limited room for improvement. Additional interventions during the follow-up period may also have affected the results. However, although slightly more participants in the comparator group than in the ERP group received additional treatment during the follow-up period, sensitivity analyses excluding participants who had received extra interventions showed no impact on the results.

From the health care organization perspective, direct intervention costs were higher for the ERP intervention than for the comparator ($15.14 per participant). From the wider health care sector perspective (direct intervention costs and other health care costs), ERP was dominant, meaning that it generated more QALYs (and more treatment responders) at a lower cost than the comparator. This has the potential of making the ERP intervention attractive for health care services providing treatment for young individuals with TS or CTD, despite the higher costs for the intervention itself, compared with psychoeducation. When broadening the focus even further to also include societal costs not related to health care (societal perspective), ERP was no longer dominant, costing more than the comparator. Nevertheless, the ICER of $18 123 was well below the considered willingness-to-pay threshold of $79 000.^41^ This ICER was comparable to the £16 708 (approximately US $20 800) cost per QALY gained reported at 18 months after randomization in the ORBIT trial.^14^ In summary, the probability of ERP being cost-effective compared with psychoeducation at the 12-month follow-up varied for each increasingly wider costing perspective: 78% for the health care organization perspective, 100% (dominance) for the health care sector perspective, and 65% for the societal perspective. A 12-month time frame is typically considered short for a health economic evaluation, and an extended time horizon has, on average, been shown to lead to more favorable estimates.^43^ This is important when the impact of health interventions may extend into the future, which is the case for interventions targeting young people’s mental health.

Given that many individuals do not access any treatment for their tic disorder and that both interventions assessed here resulted in clinically relevant within-group improvements, without significant between-group differences in the primary outcome, implementation of either intervention into regular clinical practice could increase the availability of evidence-based treatment. However, given that the sister ORBIT trial^13^ did find that ERP was superior to psychoeducation in reducing tic severity and that both the ORBIT and the current trial found that ERP was more cost-effective than psychoeducation, particularly from a health care sector perspective, we would recommend the preferential implementation of ERP.

### Strengths and Limitations

The strengths of this study include nationwide recruitment of a large sample, the use of an active comparator, thorough and transparent masking procedures, external monitoring, low attrition, and the maintenance of an experimental control throughout the 12-month follow-up. Although participants may have tried other interventions during the follow-up, they were not systematically crossed over to ERP, reducing the risk of contaminated follow-up data.

Study limitations include a somewhat restricted external validity due to a population sample with relatively mild symptom severity and the exclusion of comorbid autism, the absence of a third waitlist group to separate the effects of treatment from the natural passage of time, the absence of measuring health economic spillover effects (eg, potential secondary effects on parents and siblings), and a relatively short time frame to determine longer-term societal costs and effects.

## Conclusions

This follow-up study of an RCT found no statistically significant changes in tic severity from the 3-month to the 12-month follow-up for either the internet-delivered ERP group or the internet-delivered psychoeducation group. Although ERP was not superior to psychoeducation alone in reducing tic severity at the end of the follow-up period, we recommend ERP for clinical implementation due to its likely cost-effectiveness and support from previous literature.

## Notes

Supplement 1.
Trial Protocol

Supplement 2. **eMethods 1.** Eligibility criteria **eMethods 2.** Masking procedures **eMethods 3.** Further details on the health economic evaluation **eTable 1.** Baseline demographics and clinical characteristics for study participants **eTable 2.** Results of complementary linear mixed models for the primary and secondary outcomes **eTable 3.** Observed medians and means at all assessment points for additional secondary outcomes **eTable 4.** Results of linear quantile mixed models for additional secondary outcomes **eTable 5.** Group allocation guesses and motivations, by assessors **eTable 6.** Health economic evaluation, baseline outcomes and costs (2021 USD) **eTable 7.** Unit costs used in the costing of resources, in 2021 USD **eTable 8.** Cost means and differences from baseline to the 12-month follow-up, after multiple imputation, in 2021 USD **eTable 9.** Mean CHU9D utility scores per assessment point and total QALYs over the study period, after multiple imputation **eTable 10.** Outcomes and costs from baseline to the 12-month follow-up, after multiple imputation **eFigure 1.** Cost-effectiveness planes with treatment response as the outcome for three costing perspectives **eFigure 2.** Cost-effectiveness acceptability curves with treatment response as the outcome for three costing perspectives **eFigure 3.** Cost-effectiveness acceptability curves with QALYs as the outcome for three costing perspectives

Supplement 3.
Data Sharing Statement
