# Supplementary material for: Internet-Delivered Exposure and Response Prevention for Pediatric Tourette Syndrome: 12-Month Follow-Up of a Randomized Clinical Trial
Source: JAMA Netw Open. 2024 May 3;7(5):e248468. doi: 10.1001/jamanetworkopen.2024.8468 (PMC11069081; doi:10.1001/jamanetworkopen.2024.8468)
Supplement: Supplement 2. — eMethods 1. Eligibility criteria eMethods 2. Masking procedures eMethods 3. Further details on the health economic evaluation eTable 1. Baseline demographics and clinical characteristics for study participants eTable 2. Results of complementary linear mixed models for the primary and secondary outcomes eTable 3. Observed medians and means at all assessment points for additional secondary outcomes eTable 4. Results of linear quantile mixed models for additional secondary outcomes eTable 5. Group allocation guesses and motivations, by assessors eTable 6. Health economic evaluation, baseline outcomes and costs (2021 USD) eTable 7. Unit costs used in the costing of resources, in 2021 USD eTable 8. Cost means and differences from baseline to the 12-month follow-up, after multiple imputation, in 2021 USD eTable 9. Mean CHU9D utility scores per assessment point and total QALYs over the study period, after multiple imputation eTable 10. Outcomes and costs from baseline to the 12-month follow-up, after multiple imputation eFigure 1. Cost-effectiveness planes with treatment response as the outcome for three costing perspectives eFigure 2. Cost-effectiveness acceptability curves with treatment response as the outcome for three costing perspectives eFigure 3. Cost-effectiveness acceptability curves with QALYs as the outcome for three costing perspectives [file jamanetwopen-e248468-s002.pdf]

Supplementary Online Content

Andrén P, Sampaio F, Ringberg H, et al. Internet-delivered exposure and response prevention for pediatric Tourette syndrome: 12-month follow-up of a randomized clinical trial. *JAMA Netw Open*. 2024;7(4):e248468. doi:10.1001/jamanetworkopen.2024.8468

eMethods 1. Eligibility criteria
eMethods 2. Masking procedures
eMethods 3. Further details on the health economic evaluation
eTable 1. Baseline demographics and clinical characteristics for study participants
eTable 2. Results of complementary linear mixed models for the primary and secondary outcomes
eTable 3. Observed medians and means at all assessment points for additional secondary outcomes
eTable 4. Results of linear quantile mixed models for additional secondary outcomes
eTable 5. Group allocation guesses and motivations, by assessors
eTable 6. Health economic evaluation, baseline outcomes and costs (2021 USD).
eTable 7. Unit costs used in the costing of resources, in 2021 USD
eTable 8. Cost means and differences from baseline to the 12-month follow-up, after multiple imputation, in 2021 USD
eTable 9. Mean CHU9D utility scores per assessment point and total QALYs over the study period, after multiple imputation
eTable 10. Outcomes and costs from baseline to the 12-month follow-up, after multiple imputation
eFigure 1. Cost-effectiveness planes with treatment response as the outcome for three costing perspectives
eFigure 2. Cost-effectiveness acceptability curves with treatment response as the outcome for three costing perspectives
eFigure 3. Cost-effectiveness acceptability curves with QALYs as the outcome for three costing perspectives
eReferences.

This supplementary material has been provided by the authors to give readers additional information about their work.

© 2024 Andrén P et al. *JAMA Network Open*.

eMethods 1. Eligibility criteria

Eligible participants were 9-to-17-year-old individuals with a DSM-5 diagnosis of TS or CTD[1] who had a Yale Global Tic Severity Score (YGTSS) Total Tic Severity Score (TTSS) >15 (or >10 if only motor *or* vocal tics had been present during the last week),[2] had at least one parent available to participate in the treatment, and had access to at least one computer and one mobile phone per family. Participants were excluded if they had received ≥8 sessions of BT for tics with a qualified therapist within the past year, were receiving simultaneous psychological treatment for TS/CTD, had initiated or adjusted any psychotropic medication for TS/CTD within the past 8 weeks, had a diagnosis of organic brain disorder, intellectual disability, autism spectrum disorder, psychosis, bipolar disorder, anorexia nervosa or alcohol/substance dependence, were an immediate risk for themselves or others requiring urgent medical attention (e.g., suicidality or self-injurious tics), were not able to read and communicate in Swedish or had a close relative already enrolled in the trial.

eMethods 2. Masking procedures

The principal investigator, outcome assessors, statistician, and health economists were blind to group allocation throughout the trial. The trial coordinator was blind only during the analysis phase through the use of dummy variables for participant ID and group allocation. Participants were instructed not to reveal information about their allocated treatment to the outcome assessor. In cases where the group allocation was accidentally revealed, a new blind assessor watched an edited video recording of the original assessment and performed a second assessment, used in the analysis. Outcome assessors were asked to guess the participants' group allocation after each assessment.

eMethods 3. Further details on the health economic evaluation

The methods of the health economic evaluation are described in the Methods section of the paper and in **Supplement 1**. In this section we present additional details.

© 2024 Andrén P et al. *JAMA Network Open*.

Estimation of QALYs

Quality-adjusted life years (QALYs) were estimated by mapping KIDSCREEN-10 scores onto the multi-attribute utility instrument Child Health Utility 9 Dimensions (CHU9D)[3] using a crosswalk algorithm.[4] This was done because KIDSCREEN-10 scores cannot be directly used to derive QALYs. The CHU9D is a generic measure of health-related quality of life across nine dimensions: worried, sad, pain, tired, annoyed, schoolwork/homework, sleep, daily routine, and activities, each with five response categories representing different levels of severity, ranging between 1 (excellent) and 5 (poor). The following algorithm was used:

CHU9D utility = 0.222655 + (0.037867*KIDSCREEN-10_item_1) + (0.023085*KIDSCREEN-10_item_2) + (0.037192*KIDSCREEN-10_item_3) + (0.021284*KIDSCREEN-10_item_4) + (0.024877*KIDSCREEN-10_item_9) + (0.022256*KIDSCREEN-10_item_10).

Total QALY gains over the trial period were estimated using the area under the curve method.[5]

Further details on costing

Costs were estimated by multiplying frequencies by unit costs (**eTable 6**). Costs for healthcare resources were estimated using pricelists[6] and estimates from the Cost per Patient Database.[7] Medication costs were estimated using market prices.[8] Productivity losses due to absenteeism from school for children were estimated by multiplying the number of days absent from school by the daily cost of a child in school. Productivity losses related to reduced efficiency at school corresponded to the number of days at school not performing fully multiplied by a weighed score representing the reduction in productivity.[9] Productivity losses due to absenteeism from paid work for parents were the product of the number of days absent from work to care for the child by the average salary in Sweden (including social fees).[10] Productivity losses related to absenteeism from unpaid work (housework) corresponded to the number of days not performing unpaid work multiplied by the cost of leisure time.[11] Total costs were calculated for each participant and aggregated over the study period (approximately 62 weeks). Costs were estimated in 2021 Swedish krona (SEK) and converted to 2021 U.S. dollar (USD) using

© 2024 Andrén P et al. *JAMA Network Open*.

Purchasing Power Parities for Gross Domestic Product.[12] A discount rate of 3% was applied to costs and QALYs, as per Swedish guidelines.

Data analysis

Multiple imputation by chained equations was employed to account for missing data, assuming the cost and health outcome data were likely to be missing at random.[13] Ten datasets were imputed. Differences in costs and QALYs between groups were examined with generalized linear models[14] to allow for the consideration of other distributions to fit the data.[14] Gamma distributions and log links were used for cost data. Gaussian distributions and identify links were used for QALY data. Total costs were analyzed controlling for baseline costs. Total QALYs were analyzed controlling for baseline CHU9D values.[15]

Non-parametric bootstrapping with 5000 iterations was carried out to deal with uncertainty around the cost and outcome data. This uncertainty is presented on cost-effectiveness planes. A cost-effectiveness plane is a cloud of the 5000 bootstrapped incremental costs and effects across four quadrants, where each quadrant has a decision implication. The probability of cost-effectiveness at different willingness-to-pay thresholds is displayed on cost-effectiveness acceptability curves (CEAC).[16] The CEAC captures decision uncertainty and shows the probability of internet-delivered exposure and response prevention being cost-effective at different cost-effectiveness thresholds.[17,18]

Data was cleaned using Microsoft Excel and data analyses were performed using Stata version 15.1.

© 2024 Andrén P et al. *JAMA Network Open*.

eTable 1. Baseline demographics and clinical characteristics for study participants

**Characteristic, *n* (%)**	**ERP (*n*=111)**	**Comparator (*n*=110)**	**Total (*N*=221)**
Age, mean (SD); range	12.03 (2.26); 9-17	12.14 (2.25); 9-17	12.09 (2.25); 9-17
Gender			
Male	71 (64.0)	81 (73.6)	152 (68.8)
Female	39 (35.1)	29 (26.4)	68 (30.8)
Other	1 (0.9)	0 (0.0)	1 (0.5)
Age of tic onset, mean (SD); range	5.68 (1.98); 2-11	6.19 (2.13); 2-14	5.93 (2.07); 2-14
Tic disorder			
Tourette syndrome	104 (93.7)	98 (89.1)	202 (91.4)
Chronic motor tic disorder	7 (6.3)	9 (8.2)	16 (7.2)
Chronic vocal tic disorder	0 (0.0)	3 (2.7)	3 (1.4)
Comorbidity			
Any comorbidity	44 (39.6)	40 (3.6)	84 (38.0)
Attention-deficit/hyperactivity disorder	20 (18.0)	14 (12.7)	34 (15.4)
Anxiety disorder	16 (14.4)	15 (13.6)	31 (14.0)
Obsessive-compulsive disorder	11 (9.9)	6 (5.5)	17 (7.7)
Depression	1 (0.9)	3 (2.7)	4 (1.8)
Other[A]	7 (6.3)	3 (2.7)	10 (4.5)
Medication status			
None	94 (84.7)	95 (86.4)	189 (85.5)
Melatonin	8 (7.2)	9 (8.6)	17 (7.7)
ADHD medication[B]	9 (8.1)	5 (4.5)	14 (6.3)
α2-agonist	4 (3.6)	1 (0.9)	5 (2.3)
SSRI	2 (1.8)	3 (2.7)	5 (2.3)
Antipsychotic	1 (0.9)	1 (0.9)	2 (0.9)
Antihistaminic	0 (0.0)	2 (1.8)	2 (0.9)
Distance to the clinic in km, mean (SD); range	220 (251); 0-1226	194 (219); 1-869	207 (235); 0-1226
Highest level of parental education[C]			
Primary school	0 (0.0)	3 (2.7)	3 (1.4)
Secondary school	17 (15.3)	19 (17.3)	36 (16.3)
College/University <2 years	12 (10.8)	6 (5.5)	18 (8.1)
College/University ≥2 years	78 (70.2)	80 (72.7)	158 (71.5)
Postgraduate education	4 (3.6)	2 (1.8)	6 (2.7)
Parental occupation[C]			
Working	101 (91.0)	104 (94.5)	205 (92.8)
Student	4 (3.6)	3 (2.7)	7 (3.2)

© 2024 Andrén P et al. *JAMA Network Open*.

Other[D]	6 (5.4)	3 (2.7)	9 (4.1)
Place of birth, parent[C]			
Sweden	101 (91.0)	104 (94.5)	205 (92.8)
Rest of Europe	7 (6.3)	1 (0.9)	8 (3.6)
Other	3 (2.7)	5 (4.5)	8 (3.6)
Place of birth, parent[E]			
Sweden	95 (85.6)	88 (80.0)	183 (82.8)
Rest of Europe	9 (8.1)	7 (6.4)	16 (7.2)
Other	7 (6.3)	14 (12.7)	21 (9.5)
Not available	0 (0.0)	1 (0.9)	1 (0.5)
Previous contact with healthcare services for TS/CTD	76 (68.5)	64 (58.2)	140 (63.3)
Previous BT for TS/CTD	12 (10.8)	9 (8.2)	21 (9.5)

Note: A= Includes dyscalculia, dyslexia, excoriation (skin-picking) disorder, gender dysphoria, hoarding disorder, language disorder, and oppositional defiant disorder; B = Includes stimulants and atomoxetine; C = Primary parent supporting the treatment; D = Unemployed, sick leave or retired; E = Second parent.

Abbreviations: ADHD = attention-deficit/hyperactivity disorder; comparator = therapist-supported internet-delivered education for children and adolescents with Tourette syndrome or chronic tic disorder; CTD = chronic tic disorder; km = kilometer; ERP = therapist-supported internet-delivered exposure with response prevention for children and adolescents with Tourette syndrome or chronic tic disorder; SD = standard deviation; SSRI = selective serotonin reuptake inhibitor; TS = Tourette syndrome.

© 2024 Andrén P et al. *JAMA Network Open*.

eTable 2. Results of complementary linear mixed models for the primary and secondary outcomes

Outcome	Intention-to-treat linear mixed models								
	Within-group analysis ERP			Within-group analysis comparator			Interaction between treatment and time		
	Coefficient (95% CI)	*p*	Effect size (95% CI)[a]	Coefficient (95% CI)	*p*	Effect size (95% CI)[a]	Coefficient (95% CI)	*p*	Effect size (95% CI)[a]
**YGTSS-TTSS**									
Baseline to 12FU	-1.72 (-1.98 to -1.47)	<.001*	1.03 (0.86 to 1.20)	-1.49 (-1.76 to -1.22)	<.001*	0.81 (0.65 to 0.97)	-0.24 (-0.61 to 0.13)	.17	0.13 (-0.10 to 0.37)
3FU to 12FU	-0.64 (-1.21 to -0.07)	.03*	0.17 (0.01 to 0.34)	-0.46 (-1.05 to -0.14)	.13	0.12 (-0.03 to 0.27)	-0.18 (-1.01 to 0.64)	.67	0.05 (-0.18 to 0.28)
**YGTSS Impairment**									
Baseline to 12FU	-2.77 (-3.15 to -2.39)	<.001*	1.31 (1.12 to 1.50)	-2.94 (-3.31 to -2.57)	<.001*	1.35 (1.15 to 1.56)	0.16 (-0.37 to 0.70)	.54	-0.08 (-0.36 to 0.21)
3FU to 12FU	-0.66 (-1.44 to 0.11)	.09	0.16 (-0.06 to 0.38)	-1.25 (-1.90 to -0.60)	<.001*	0.30 (0.13 to 0.47)	0.59 (-0.44 to 1.60)	.26	-0.14 (-0.42 to 0.14)
**CGI-S**									
Baseline to 12FU	-0.26 (-0.30 to -0.23)	<.001*	1.18 (0.99 to 1.36)	-0.23 (-0.27 to -0.19)	<.001*	0.95 (0.73 to 1.17)	-0.03 (-0.09 to 0.02)	.20	0.15 (-0.13 to 0.43)
3FU to 12FU	-0.14 (-0.21 to -0.07)	<.001*	0.30 (0.13 to 0.48)	-0.11 (-0.20 to -0.03)	.01*	0.22 (0.06 to 0.38)	-0.03 (-0.14 to 0.08)	.63	0.06 (-0.19 to 0.30)
**PTQ**									
Baseline to 12FU	-3.21 (-3.67 to -2.75)	<.001*	0.93 (0.76 to 1.09)	-2.99 (-3.59 to -2.39)	<.001*	0.75 (0.57 to 0.93)	-0.22 (-0.97 to 0.54)	.57	0.06 (-0.20 to 0.31)
3FU to 12FU	-1.42 (-2.68 to -0.16)	.03*	0.42 (0.01 to 0.83)	-1.22 (-2.98 to 0.54)	.18	0.33 (-0.16 to 0.82)	-0.24 (-2.38 to 1.90)	.83	0.07 (-0.56 to 0.70)
**C&A-GTS-QOL**									
Baseline to 12FU	-1.84 (-2.41 to -1.27)	<.001*	0.46 (0.31 to 0.60)	-2.31 (-2.86 to -1.76)	<.001*	0.57 (0.42 to 0.73)	0.47 (-0.32 to 1.27)	.24	-0.12 (-0.33 to 0.09)
3FU to 12FU	0.49 (-0.66 to 1.64)	.41	-0.06 (-0.23 to 0.11)	0.32 (-0.65 to 1.30)	.52	-0.04 (-0.16 to 0.08)	0.19 (-1.34 to 1.71)	.81	-0.02 (-0.22 to 0.18)
**CGAS**									
Baseline to 12FU	1.84 (1.55 to 2.12)	<.001*	0.88 (0.69 to 1.06)[b]	1.38 (1.11 to 1.65)	<.001*	0.70 (0.50 to 0.90)[b]	0.46 (0.07 to 0.85)	.02*	0.23 (-0.04 to 0.49)[b]
3FU to 12FU	0.56 (-0.01 to 1.14)	.06	0.13 (-0.03 to 0.28)[b]	0.72 (0.22 to 1.23)	.005*	0.17 (0.04 to 0.30)[b]	-0.16 (-0.93 to 0.61)	.68	0.15 (-0.25 to 0.18)[b]
**OCI-CV**									
Baseline to 12FU	-0.34 (-0.57 to -0.10)	.005*	0.22 (0.03 to 0.41)	-0.43 (-0.62 to -0.23)	<.001*	0.29 (0.14 to 0.43)	0.09 (-0.22 to 0.40)	.57	-0.06 (-0.29 to 0.17)

© 2024 Andrén P et al. *JAMA Network Open*.

3FU to 12FU	0.47 (0.01 to 0.93)	.05*	-0.15 (-0.32 to 0.02)	0.00 (-0.39 to 0.39)	>.99	0.00 (-0.14 to 0.14)	0.47 (-0.14 to 1.08)	.13	-0.15 (-0.37 to 0.06)
**SMFQ-C**									
Baseline to 12FU	-0.15 (-0.27 to -0.03)	.01*	0.17 (0.03 to 0.32)	-0.25 (-0.38 to -0.12)	<.001*	0.33 (0.10 to 0.56)	0.09 (-0.08 to 0.27)	.30	-0.11 (-0.38 to 0.15)
3FU to 12FU	0.03 (-0.35 to 0.41)	.87	-0.01 (-0.22 to 0.19)	0.00 (-0.27 to 0.28)	.98	-0.00 (-0.16 to 0.15)	0.03 (-0.44 to 0.51)	.89	-0.02 (-0.28 to 0.25)
**SMFQ-P**									
Baseline to 12FU	-0.29 (-0.40 to -0.18)	<.001*	0.38 (0.22 to 0.53)	-0.27 (-0.39 to -0.16)	<.001*	0.40 (0.19 to 0.61)	-0.02 (-0.18 to 0.14)	.83	0.02 (-0.24 to 0.29)
3FU to 12FU	-0.09 (-0.41 to 0.23)	.58	0.05 (-0.13 to 0.22)	-0.08 (-0.36 to 0.21)	.61	0.05 (-0.11 to 0.20)	-0.02 (-0.45 to 0.42)	.95	0.01 (-0.23 to 0.25)
**KIDSCREEN-10, child v.**									
Baseline to 12FU	0.02 (-0.18 to 0.22)	.86	0.01 (-0.14 to 0.16)[b]	0.04 (-0.16 to 0.25)	.69	0.03 (-0.14 to 0.20)[b]	-0.02 (-0.31 to 0.27)	.88	-0.02 (-0.25 to 0.21)[b]
3FU to 12FU	-0.31 (-0.77 to 0.16)	.19	-0.10 (-0.26 to 0.06)[b]	0.03 (-0.39 to 0.46)	.88	0.01 (-0.15 to 0.18)[b]	-0.34 (-0.98 to 0.29)	.29	-0.12 (-0.37 to 0.13)[b]
**KIDSCREEN-10, parent v.**									
Baseline to 12FU	0.11 (-0.06 to 0.27)	.19	0.09 (-0.07 to 0.26)[b]	-0.14 (-0.33 to 0.04)	.13	-0.13 (-0.32 to 0.06)[b]	0.25 (0.01 to 0.50)	.04*	0.23 (-0.03 to 0.48)[b]
3FU to 12FU	-0.31 (-0.66 to 0.04)	.08	-0.13 (-0.28 to 0.01)[b]	-0.27 (-0.65 to 0.10)	.16	-0.12 (-0.29 to 0.04)[b]	-0.04 (-0.55 to 0.47)	.88	-0.02 (-0.24 to 0.20)[b]

Note: a = Bootstrapped Cohen's *d* effect sizes are derived from the linear mixed models; b = The effect size has been inverted, so that an increase on this outcome measure is equivalent to a positive effect size, and vice versa; * = Significant at an alpha level of .05.

Abbreviations: 3FU: follow-up 3 months post-treatment (the primary endpoint); 12FU: follow-up 12 months post-treatment; C&A-GTS-QOL = Child and Adolescent Gilles de la Tourette Syndrome–Quality of life scale; CGAS = Children's Global Assessment Scale; CGI-S = Clinical Global Impression – Severity scale; CI = confidence interval; ERP = therapist-supported internet-delivered exposure with response prevention for children and adolescents with Tourette syndrome or chronic tic disorder; OCI-CV = Obsessive-Compulsive Inventory – Child version; PTQ = Parent Tic Questionnaire; SMFQ-C = Short Mood and Feelings Questionnaire – Child version; SMFQ-P = Short Mood and Feelings Questionnaire – Parent version; v = version; YGTSS = Yale Global Tic Severity Scale; YGTSS-TTSS = Yale Global Tic Severity Scale – Total Tic Severity Score (primary outcome).

© 2024 Andrén P et al. *JAMA Network Open*.

eTable 3. Observed medians and means at all assessment points for additional secondary outcomes

Outcome	ERP (*n*=111)		Comparator (*n*=110)	
	Median (IQR)[a]	Mean (SD)[a]	Median (IQR)[a]	Mean (SD)[a]
**CGAS**				
Baseline (*n*=221)	60 (55 to 65)	60.60 (6.59)	61 (56 to 65)	60.71 (6.46)
Post-treatment (*n*=213)	63.5 (59 to 70)	64.21 (7.90)	62 (59 to 70)	64.01 (7.59)
3-month follow-up[b] (*n*=216)	65 (60 to 72.5)	66.83 (8.64)	65 (59 to 70.5)	65.27 (7.44)
6-month follow-up (*n*=210)	69 (61 to 75)	67.92 (8.93)	65 (59 to 72)	65.84 (8.67)
12-month follow-up (*n*=208)	69 (60 to 75)	67.93 (9.12)	65 (60 to 75)	67.00 (9.40)
**OCI-CV**				
Baseline (*n*=221)	8 (4 to 13)	8.93 (6.43)	7 (3 to 12)	7.91 (5.78)
Post-treatment (*n*=211)	5 (2 to 9)	6.35 (5.75)	5 (1.5 to 11)	6.56 (5.82)
3-month follow-up[b] (*n*=207)	5 (2 to 9)	6.32 (5.87)	3.5 (1 to 10)	6.04 (6.27)
6-month follow-up (*n*=195)	6 (2 to 10)	6.51 (5.45)	4 (2 to 10)	6.29 (6.10)
12-month follow-up (*n*=194)	5.5 (2 to 10)	7.29 (7.14)	4 (1 to 10)	5.92 (5.88)
**SMFQ-C**				
Baseline (*n*=221)	4 (1 to 6)	4.55 (4.32)	3 (1 to 7)	4.53 (4.63)
Mid-treatment (*n*=212)	3 (1 to 5)	3.72 (3.87)	2 (1 to 5)	3.49 (3.61)
Post-treatment (*n*=211)	2 (0 to 4)	3.16 (4.44)	2 (0 to 5)	3.16 (3.52)
3-month follow-up[b] (*n*=207)	2 (1 to 5)	3.65 (4.51)	2 (1 to 4)	3.05 (3.29)
6-month follow-up (*n*=195)	2 (0 to 4)	2.97 (3.99)	2 (1 to 4)	3.26 (3.96)
12-month follow-up (*n*=194)	2 (0 to 6)	3.74 (5.03)	2 (0.5 to 4.5)	2.93 (3.43)
**SMFQ-P**				
Baseline (*n*=221)	3 (1 to 6)	4.44 (4.11)	3 (1 to 6)	4.21 (4.02)
Mid-treatment (*n*=210)	2 (1 to 4)	3.23 (3.92)	2 (1 to 5)	3.43 (3.67)
Post-treatment (*n*=214)	1.5 (0 to 4)	2.76 (3.80)	2 (0 to 4)	2.69 (3.02)
3-month follow-up[b] (*n*=210)	2 (0 to 4)	2.74 (3.66)	2 (0 to 4)	2.62 (3.07)

© 2024 Andrén P et al. *JAMA Network Open*.

6-month follow-up (*n*=206)	1 (0 to 4)	2.93 (4.19)	2 (0 to 4)	3.16 (3.65)
12-month follow-up (*n*=203)	1 (0 to 4)	2.61 (3.34)	2 (0 to 3)	2.43 (2.76)
**KIDSCREEN-10, child version**				
Baseline (*n*=221)	41 (37 to 44)	40.28 (5.29)	41 (37 to 44)	40.54 (5.28)
Post-treatment (*n*=211)	42 (38 to 46)	41.42 (5.71)	42 (38 to 45)	40.70 (5.91)
3-month follow-up[b] (*n*=207)	42 (37 to 46)	40.96 (6.26)	41 (37 to 44)	40.60 (5.58)
6-month follow-up (*n*=195)	43 (39 to 46)	41.65 (5.88)	41 (38 to 45)	40.74 (5.42)
12-month follow-up (*n*=194)	40.5 (37 to 45)	40.33 (6.23)	41.5 (37 to 44)	40.91 (4.98)
**KIDSCREEN-10, parent version**				
Baseline (*n*=221)	41 (37 to 43)	39.96 (4.42)	40 (37 to 43)	40.28 (4.31)
Post-treatment (*n*=214)	41 (37 to 43)	40.17 (4.87)	41 (38 to 44)	40.76 (4.27)
3-month follow-up[b] (*n*=210)	42 (38 to 45)	41.19 (4.62)	41 (38 to 44)	40.45 (4.88)
6-month follow-up (*n*=206)	41 (38 to 44)	40.38 (4.72)	41 (37 to 43)	40.32 (4.19)
12-month follow-up (*n*=203)	41 (37 to 44)	40.46 (4.77)	40 (38 to 42)	39.92 (4.00)

Note: a = Observed values calculated from completer data; b = Primary endpoint.

Abbreviations: CGAS = Children's Global Assessment Scale; ERP = therapist-supported internet-delivered exposure with response prevention for children and adolescents with Tourette syndrome or chronic tic disorder; IQR = interquartile range; Mid-treatment = 5 weeks into the treatment; OCI-CV = Obsessive-Compulsive Inventory – Child version; SMFQ-C = Short Mood and Feelings Questionnaire – Child version; SMFQ-P = Short Mood and Feelings Questionnaire – Parent version; SD = standard deviation.

© 2024 Andrén P et al. *JAMA Network Open*.

eTable 4. Results of linear quantile mixed models for additional secondary outcomes

Outcome	Intention-to-treat linear quantile mixed models								
	Within-group analysis ERP			Within-group analysis comparator			Interaction between treatment and time		
	Coefficient (95% CI)	*p*	Effect size (95% CI)[a]	Coefficient (95% CI)	*p*	Effect size (95% CI)[a]	Coefficient (95% CI)	*p*	Effect size (95% CI)[a]
**CGAS**									
Baseline to 12FU	1.86 (1.33 to 2.39)	<.001*	0.59 (0.40 to 0.78)[b]	1.25 (0.78 to 1.71)	<.001*	0.42 (0.25 to 0.58)[b]	0.66 (-0.19 to 1.52)	.13	0.23 (0.01 to 0.45)[b]
3FU to 12FU	0.85 (-0.01 to 1.71)	.05	0.13 (-0.00 to 0.26)[b]	0.29 (-0.75 to 1.33)	.58	0.04 (-0.08 to 0.17)[b]	0.76 (-0.41 to 1.94)	.20	0.12 (-0.06 to 0.29)[b]
**OCI-CV**									
Baseline to 12FU	-0.36 (-0.60 to -0.12)	.003*	0.18 (0.01 to 0.35)	-0.44 (-0.63 to -0.26)	<.001*	0.22 (0.08 to 0.36)	0.21 (-0.22 to 0.64)	.34	-0.10 (-0.30 to 0.09)
3FU to 12FU	0.00 (-0.31 to 0.31)	>.99	0.00 (-0.16 to 0.16)	0.00 (-0.35 to 0.35)	>.99	0.00 (-0.13 to 0.13)	0.53 (-0.04 to 1.10)	.07	-0.13 (-0.32 to 0.05)
**SMFQ-C**									
Baseline to 12FU	-0.31 (-0.41 to -0.22)	<.001*	0.39 (0.11 to 0.67)	-0.33 (-0.50 to -0.16)	<.001*	0.41 (0.13 to 0.70)	0.01 (-0.14 to 0.17)	.86	-0.02 (-0.33 to 0.29)
3FU to 12FU	-0.15 (-0.41 to 0.11)	.27	0.07 (-0.18 to 0.33)	-0.22 (-0.56 to 0.12)	.21	0.11 (-0.09 to 0.31)	-0.00 (-0.41 to 0.41)	.99	0.00 (-0.24 to 0.25)
**SMFQ-P**									
Baseline to 12FU	-0.28 (-0.38 to -0.17)	<.001*	0.35 (0.11 to 0.58)	-0.31 (-0.56 to -0.05)	.02*	0.38 (0.12 to 0.65)	0.02 (-0.13 to 0.17)	.80	-0.02 (-0.29 to 0.24)
3FU to 12FU	0.00 (-0.23 to 0.23)	>.99	0.00 (-0.28 to 0.28)	-0.01 (-0.28 to 0.27)	.95	-0.00 (0.14 to -0.15)	0.11 (-0.26 to 0.49)	.55	-0.06 (-0.26 to 0.14)
**KIDSCREEN-10, child v.**									
Baseline to 12FU	0.00 (-0.20 to 0.20)	>.99	0.00 (-0.17 to 0.17)[b]	0.11 (-0.09 to 0.31)	.29	0.06 (-0.08 to 0.20)[b]	-0.15 (-0.59 to 0.30)	.52	-0.09 (-0.36 to 0.17)[b]
3FU to 12FU	-0.72 (-1.64 to 0.21)	.13	-0.18 (-0.36 to 0.00)[b]	0.01 (-0.44 to 0.45)	.98	0.00 (-0.13 to 0.13)[b]	-0.50 (-1.42 to 0.42)	.29	-0.13 (-0.36 to 0.10)[b]
**KIDSCREEN-10, parent v.**									
Baseline to 12FU	0.03 (-0.23 to 0.28)	.85	0.02 (-0.18 to 0.21)[b]	-0.26 (-0.59 to 0.07)	.13	-0.19 (-0.39 to 0.02)[b]	0.22 (-0.06 to 0.50)	.12	0.15 (-0.12 to 0.42)[b]
3FU to 12FU	-0.49 (-1.09 to 0.11)	.11	-0.16 (-0.37 to 0.04)[b]	-0.42 (-0.98 to 0.14)	.14	-0.14 (-0.32 to 0.04)[b]	-0.19 (-0.85 to 0.48)	.58	-0.06 (-0.29 to 0.17)[b]

Note: a = Bootstrapped effect sizes, interpreted as differences in median relative the interquartile range, are derived from the linear quantile mixed models; b = The effect size has been inverted, so that an increase on this outcome measure is equivalent to a positive effect size, and vice versa; * = Significant at an alpha level of .05.

© 2024 Andrén P et al. *JAMA Network Open*.

Abbreviations: 3FU: follow-up 3 months post-treatment (the primary endpoint); 12FU: follow-up 12 months post-treatment; CGAS = Children's Global Assessment Scale; CI = confidence interval; ERP = therapist-supported internet-delivered exposure with response prevention for children and adolescents with Tourette syndrome or chronic tic disorder; OCI-CV = Obsessive-Compulsive Inventory – Child version; SMFQ-C = Short Mood and Feelings Questionnaire – Child version; SMFQ-P = Short Mood and Feelings Questionnaire – Parent version; v = version.

© 2024 Andrén P et al. *JAMA Network Open*.

eTable 5. Group allocation guesses and motivations, by assessors.

	6-month follow-up		12-month follow-up	
	**Frequency**	**Percentage**	**Frequency**	**Percentage**
**Correctly guessed group allocation**	113	54.9%	115	56.4%
**Correctly guessed ERP group**	62	30.0%	69	33.8%
Motivation/reason for guess				
The participant improved	40	64.5%	49	71.0%
Pure guess	21	33.9%	20	29.0%
The participant did not improve	1	1.6%	-	-
**Correctly guessed the comparator**	51	24.8%	46	22.5%
Motivation/reason for guess				
Pure guess	26	51.0%	32	69.6%
The participant did not improve	22	43.1%	12	26.1%
Other specified reason	3	5.9%	2	4.3%
**Incorrectly guessed ERP group**	51	24.8%	54	26.5%
Motivation/reason for guess				
The participant improved	26	51.0%	42	77.8%
Pure guess	24	47.1%	12	22.2%
Other specified reason	1	2.0%	-	-
**Incorrectly guessed the comparator**	42	20.4%	35	17.2%
Motivation/reason for guess				
Pure guess	28	66.7%	21	60.0%
The participant did not improve	13	31.0%	11	31.4%
Other specified reason	1	2.4%	3	8.6%

Abbreviations: ERP = therapist-supported internet-delivered exposure with response prevention for children and adolescents with Tourette syndrome or chronic tic disorder.

© 2024 Andrén P et al. *JAMA Network Open*.

eTable 6. Health economic evaluation, baseline outcomes and costs (2021 USD)

Outcomes and costs	ERP (*n*=111)	Comparator (*n*=109)[a]
	Mean (SD)	Mean (SD)
**Outcomes**		
KIDSCREEN-10, child version	39.96 (4.42)	40.21 (4.27)
CHU9D (Utility)[b]	0.869 (0.105)	0.872 (0.098)
**Costs**[c]		
Total healthcare costs	505.74 (522.88)	537.81 (668.83)
Total societal costs	1167.81 (1767.46)	879.86 (1435.86)

Note: a = One participant in the comparator was excluded from the health economic evaluation due to having been hospitalized (sometime between baseline and the primary endpoint) following a treatment-unrelated serious adverse event. Cost estimates for this hospitalization was uncertain and it was judged that the inclusion of these costs could have skewed the between-group comparison; b = CHU9D utilities were estimated from KIDSCREEN-10 (child-reported version) scores using a mapping algorithm; c = Costs refer to a 3-month timeframe prior to the participants were recruited to the study, hence excluding the costs for the study interventions. Costs are uprated to 2021 and converted from SEK to USD, where relevant, using Purchasing Power Parities.[12]

Abbreviations: CHU9D = Child Health Utility 9D; ERP = therapist-supported internet-delivered exposure with response prevention for children and adolescents with Tourette syndrome or chronic tic disorder; SD = standard deviation.

© 2024 Andrén P et al. *JAMA Network Open*.

eTable 7. Unit costs used in the costing of resources, in 2021 USD

Resource item	Unit cost (USD)[a]	Source
**Healthcare resources (per visit)**		
Counsellor	229.25	Region Stockholm and Sweden's Municipalities and Regions[b]
Dietician	313.38	Sweden's Municipalities and Regions
General practitioner	203.27	Region Stockholm
Nurse	205.76	Region Stockholm and Sweden's Municipalities and Regions[b]
Speech and language therapist	336.87	Sweden's Municipalities and Regions
Specialist practitioner[c]	477.17	Sweden's Municipalities and Regions
Physiotherapist	195.82	Sweden's Municipalities and Regions
Psychologist	272.39	Region Stockholm and Sweden's Municipalities and Regions[b]
Other treatments (e.g., acupuncture, osteopathy)	67.76	Market prices
**Medication**		
Over the counter supplements	Individual product prices	Market price from the Swedish pharmacy chain Apotea[8]
Prescription drugs	Individual product prices	The Dental and Pharmaceutical Benefits Agency of Sweden
**Support and assistance**		
Host family (per day)	80.07	Sweden's Municipalities and Regions
Special assistant (per hour)	35.57	Swedish Insurance Agency
Special teacher (per hour)	45.06	Own estimate
Study buddy (per hour)	51.71	Own estimate
**Productivity losses**		
Average wage/hour in Sweden[d]	36.53	Statistics Sweden
Cost of leisure time/hour[d]	17.84	Post-tax wage/hour in Sweden[11]
Cost per child/day at school	79.72	Own estimate based on Swedish National Agency for Education
**Intervention cost**		
Internet-delivered BT therapist average wage (per hour)	36.96	Project documentation

© 2024 Andrén P et al. *JAMA Network Open*.

Note: a = Costs are uprated to 2021 and converted from SEK to USD, where relevant, using Purchasing Power Parities;[12] b = Corresponds to an average of the cost for primary care and specialist care; c = Based on an average of 11 medical specialties; d = Includes social fees of 43.3%.[19]

© 2024 Andrén P et al. *JAMA Network Open*.

eTable 8. Cost means and differences from baseline to the 12-month follow-up,[a] after multiple imputation, in 2021 USD

	ERP (*n*=111)		Comparator (*n*=109)[b]		Mean difference			
**Type of cost, from the TiC-P**	Mean	SE	Mean	SE	Unadjusted mean diff.	Adjusted mean diff.[c]	95% LCI	95% UCI
Healthcare visits	2043.37	433.04	2685.79	434.85	-642.42	-550.93	-782.99	505.81
Medication/supplements	278.79	86.48	381.88	94.27	-103.09	-70.56	-136.10	359.12
Social support and assistance	936.07	427.21	1135.70	385.65	-199.63	-164.80	-356.74	2322.36
Parental paid productivity loss[d]	2119.33	555.37	1765.81	343.19	353.51	164.81	-321.82	2090.91
Parental unpaid productivity loss[e]	973.28	277.95	1891.08	393.94	-917.81	-773.92	-683.90	-253.54
School support	1246.04	712.69	686.49	285.14	559.55	457.01	-122.14	7996.41
Child school absenteeism	1449.82	209.74	1601.57	169.89	-151.75	-153.94	-331.10	248.61
Child school presenteeism	338.35	129.88	441.93	125.33	-103.58	-205.64	-164.11	-61.56
**Costs summed per perspective**								
Intervention costs (healthcare organization perspective)[f]	117.38	8.78	102.23	3.65	15.14	-	5.08	25.20
Total healthcare costs (healthcare sector perspective)[g]	2440.12	460.12	3176.10	454.17	-735.98	-84.48[i]	-440.20[i]	977.60[i]
Total societal costs (societal perspective)[h]	9515.69	1712.88	10734.37	1406.48	-1218.68	127.66[i]	-1061.62[i]	2562.26[i]

Note: a = The TiC-P data in eTable 7 correspond to costs related to resource use estimated and summed over the periods of post-treatment, 3-, 6- and 12-month follow-ups. The 3-month recall time frame of the TiC-P was approximately equivalent to the period between baseline and post-treatment (10 weeks), post-treatment and the 3-month follow-up (3 months), and the 3- and 6-month follow-ups (3 months). For the period between the 6- and the 12-month follow-ups, linear consumption was assumed, hence total costs for this period were multiplied by two. b = One participant in the comparator was excluded from the health economic evaluation due to having been hospitalized (sometime between baseline and the primary endpoint) following a treatment-unrelated serious adverse event. Cost estimates for this hospitalization was uncertain and it was judged that the inclusion of these costs could have skewed the between-group comparison; c = Adjusted mean differences calculated using generalized linear models (GLM) adjusted for baseline cost, except for the intervention costs; d = Work absenteeism; e = Housework; f = Costs of the ERP or comparator interventions; g = Costs of the ERP or comparator interventions, healthcare visits, and medication/supplements; h = Costs of the ERP or comparator interventions, healthcare visits, medication/supplements, and societal costs (e.g., productivity losses, child school absenteeism); i = Bootstrapped adjusted values.

© 2024 Andrén P et al. *JAMA Network Open*.

Abbreviations: ERP = therapist-supported internet-delivered exposure with response prevention for children and adolescents with Tourette syndrome or chronic tic disorder; LCI = lower 95% confidence interval; SE = standard error; TiC-P = Trimbos/iMTA questionnaire for costs associated with psychiatric illness; UCI = upper 95% confidence interval.

© 2024 Andrén P et al. *JAMA Network Open*.

eTable 9. Mean CHU9D utility scores per assessment point and total QALYs over the study period, after multiple imputation

Outcome	ERP (*n*=111)			Comparator (*n*=109)[a]		
	Mean	SE	95% CI	Mean	SE	95% CI
**CHU9D** (Utility)[b]						
Baseline	0.869	0.010	0.850-0.889	0.873	0.009	0.854-0.891
Post-treatment	0.894	0.010	0.874-0.914	0.879	0.010	0.859-0.898
3-month follow-up	0.877	0.011	0.855-0.898	0.872	0.010	0.852-0.892
6-month follow-up	0.885	0.011	0.863-0.907	0.861	0.012	0.836-0.886
12-month follow-up	0.874	0.011	0.852-0.896	0.865	0.012	0.841-0.889
**Total QALYs undiscounted (baseline to 12-month follow-up)**	1.052	0.010	1.031-1.073	1.036	0.010	1.016-1.055
**Total QALYs discounted at 3% (baseline to 12-month follow-up)**	1.016	0.010	0.996-1.036	1.000	0.009	0.981-1.019

Note: a = One participant in the comparator was excluded from the health economic evaluation due to having been hospitalized (sometime between baseline and the primary endpoint) following a treatment-unrelated serious adverse event. Cost estimates for this hospitalization was uncertain and it was judged that the inclusion of these costs could have skewed the between-group comparison; b = CHU9D utilities were estimated from KIDSCREEN-10 (child-reported version) scores using a mapping algorithm.

Abbreviations: CHU9D = Child Health Utility 9D; CI = confidence interval; ERP = therapist-supported internet-delivered exposure with response prevention for children and adolescents with Tourette syndrome or chronic tic disorder; QALY = quality-adjusted life year; SE = standard error.

© 2024 Andrén P et al. *JAMA Network Open*.

eTable 10. Outcomes and costs from baseline to the 12-month follow-up,[a] after multiple imputation

	ERP (*n*=111)		Comparator (*n*=109)[b]		Mean difference					
**Outcomes**	Mean	SE	Mean	SE	Unadjusted mean diff.	Adjusted mean diff.[c d]	95% LCI	95% UCI	ICER Treatment response[d]	ICER QALY[d]
Total QALYs	1.016	0.027	1.000	0.013	0.016	0.007	-0.013	0.027	-	-
Treatment response[E]	0.551	-	0.500	-	0.051	-	-0.085	0.187	-	-
**Costs summed per perspective**[F]										
Intervention costs (healthcare organization perspective)[g]	117.38	8.78	102.23	3.65	15.14	-	5.08	25.20	295	2150
Total healthcare costs (healthcare sector perspective)[h]	2440.12	460.12	3176.10	454.17	-735.98	-84.48	-440.20	977.60	Dominant[j]	Dominant[j]
Total societal costs (societal perspective)[i]	9515.69	1712.88	10734.37	1406.48	-1218.68	127.66	-1061.62	2562.26	2484	18123

Note: a = The TiC-P data in eTable 7 correspond to costs related to resource use estimated and summed over the periods of post-treatment, 3-, 6- and 12-month follow-ups. The 3-month recall time frame of the TiC-P was approximately equivalent to the period between baseline and post-treatment (10 weeks), post-treatment and the 3-month follow-up (3 months), and the 3- and 6-month follow-ups (3 months). For the period between the 6- and the 12-month follow-ups, linear consumption was assumed, hence total costs for this period were multiplied by two. b = One participant in the comparator was excluded from the health economic evaluation due to having been hospitalized (sometime between baseline and the primary endpoint) following a treatment-unrelated serious adverse event. Cost estimates for this hospitalization was uncertain and it was judged that the inclusion of these costs could have skewed the between-group comparison; c = Adjusted mean differences calculated using generalized linear models (GLM) adjusted for baseline CHU9D scores in the analysis of QALYs and baseline cost in the analyses of costs (except for the intervention costs); d = Bootstrapped adjusted values; e = Treatment response is defined as a score of 1 ("Very much improved") or 2 ("Much improved") on the Clinical Global Impression – Improvement (CGI-I) scale; f = Costs are uprated to 2021 and converted from SEK to USD, where relevant, using Purchasing Power Parities;[12] g = Costs of the ERP or comparator interventions; h = Costs of the ERP or comparator interventions, healthcare visits, and medication/supplements; i = Costs of the ERP or comparator interventions, healthcare visits, medication/supplements, and societal costs (e.g., productivity losses, child school absenteeism). j = Dominant means that ERP dominated the comparator, yielding more health benefits (more QALYs and more treatment responders) and lower costs than the comparator.

© 2024 Andrén P et al. *JAMA Network Open*.

Abbreviations: CHU9D = Child Health Utility 9D; ERP = therapist-supported internet-delivered exposure with response prevention for children and adolescents with Tourette syndrome or chronic tic disorder; ICER = incremental cost-effectiveness ratio; LCI = lower 95% confidence interval; QALY = quality-adjusted life year; SE = standard error; UCI = upper 95% confidence interval.

© 2024 Andrén P et al. *JAMA Network Open*.

eFigure 1. Cost-effectiveness planes with treatment response as the outcome for three costing perspectives.

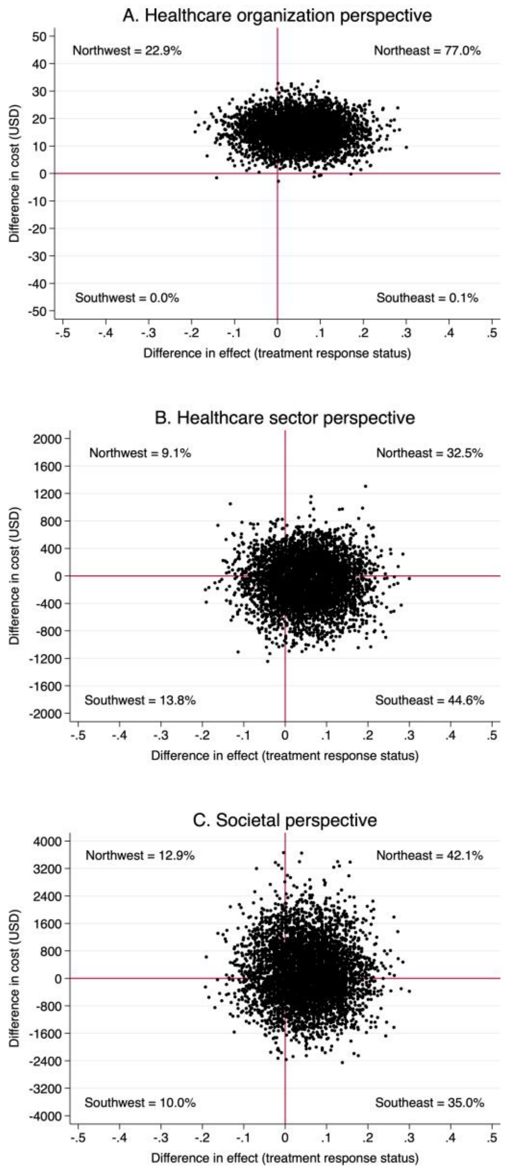

Note: All three cost-effectiveness planes compare ERP to the comparator using treatment response status as the outcome. Treatment response is defined as a score of 1 (“Very much improved”) or 2 (“Much improved”) on the Clinical Global Impression – Improvement (CGI-I) scale. The three planes only differ by the costing perspective. The healthcare organization perspective (A) includes costs of the ERP or comparator interventions (i.e., the therapist-support time). The healthcare sector perspective (B) includes costs of the ERP or comparator interventions, healthcare visits, and medication/supplements. The societal perspective (C) includes costs of the ERP or comparator interventions, healthcare visits, medication/supplements, and other sector costs (e.g., productivity losses, child school absenteeism). The probability of the ERP group showing higher treatment response rates at higher costs (northeast quadrant) is 77.0% in A, 32.5% in B, and 42.1% in C. The equivalent

© 2024 Andrén P et al. *JAMA Network Open*.

probability of the ERP group showing higher treatment response rates at lower costs (southeast quadrant) is 0.1% in A, 44.6% in B, and 35.0% in C. The probability of the comparator showing higher treatment response rates at higher costs (northwest quadrant) is 22.9% in A, 9.1% in B, and 12.9% in C. The equivalent probability of the comparator group showing higher treatment response rates at lower costs (southwest quadrant) is 0.0% in A, 13.8% in B, and 10.0% in C.

Abbreviations: ERP = therapist-supported internet-delivered exposure with response prevention for children and adolescents with Tourette syndrome or chronic tic disorder.

© 2024 Andrén P et al. *JAMA Network Open*.

eFigure 2. Cost-effectiveness acceptability curves with treatment response as the outcome for three costing perspectives

A. Healthcare organization perspective

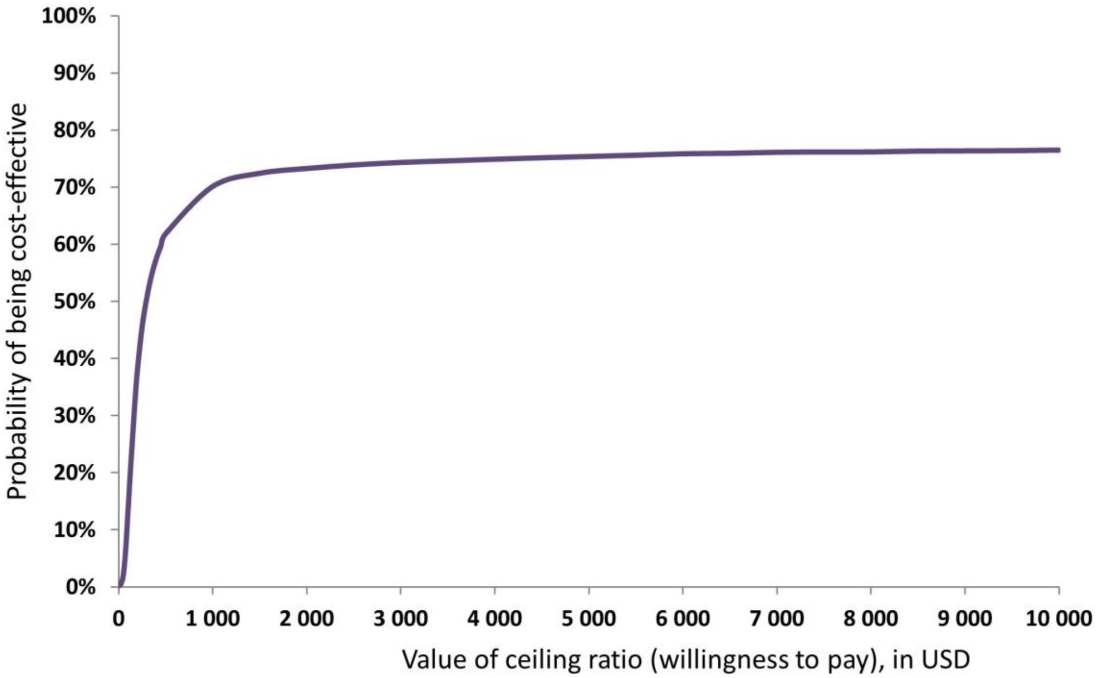

© 2024 Andrén P et al. *JAMA Network Open*.

B. Healthcare sector perspective

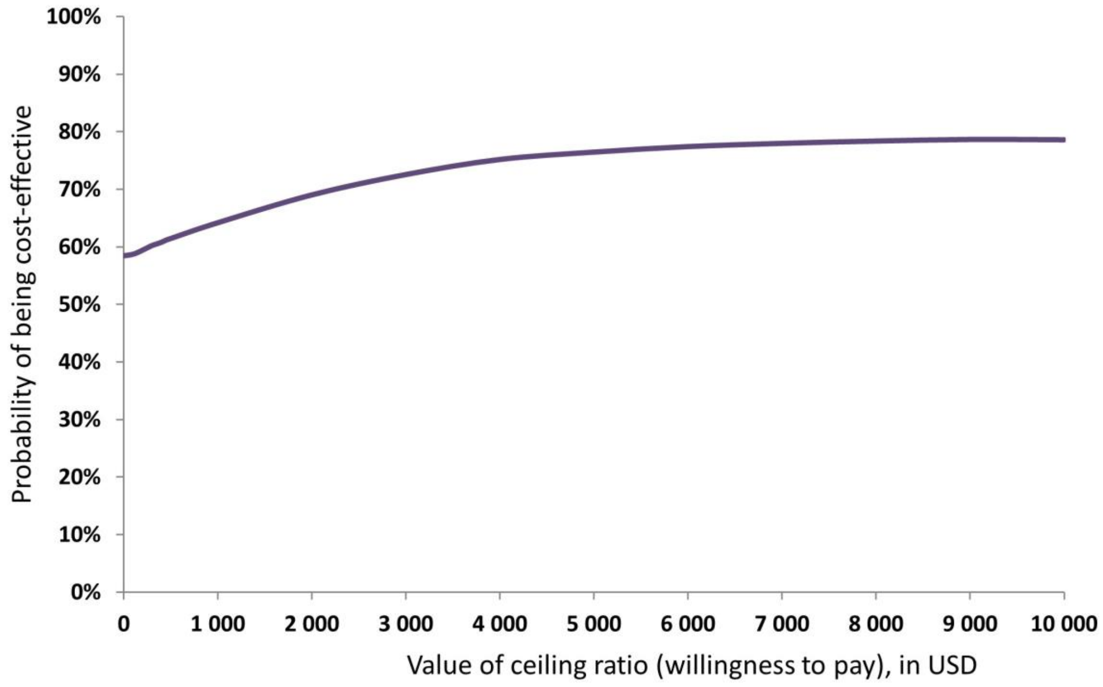

© 2024 Andrén P et al. *JAMA Network Open*.

C. Societal perspective

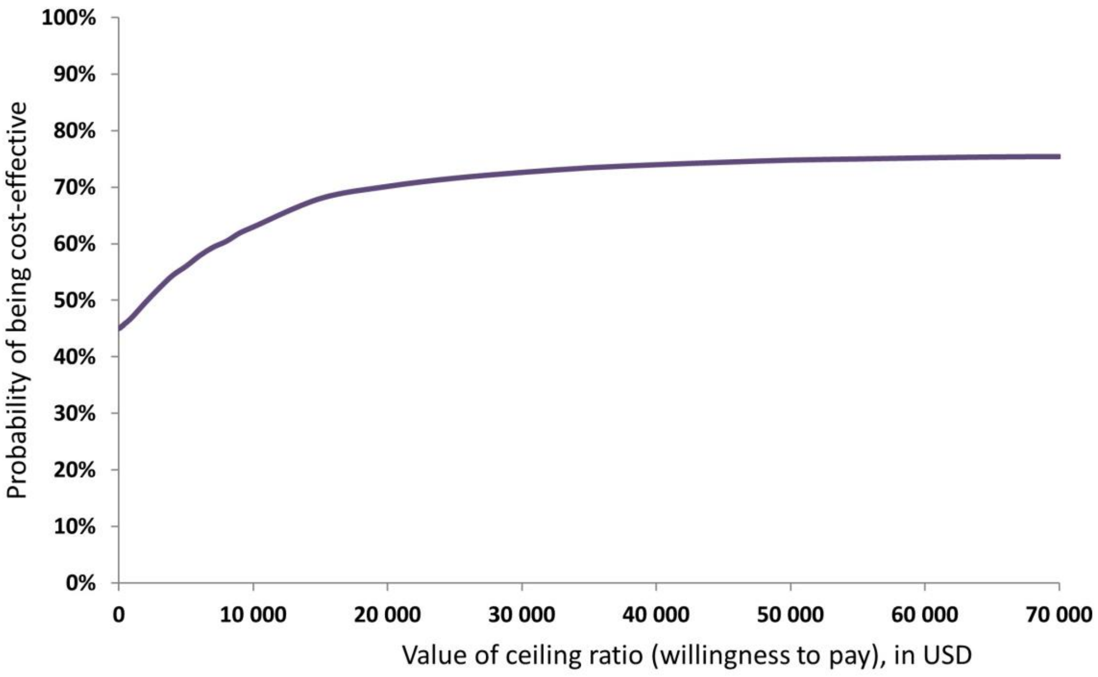

Note: The cost-effectiveness acceptability curves show the probability for the ERP intervention to be cost-effective at different willingness-to-pay levels (in USD), with treatment response as the outcome, for three costing perspectives. Treatment response is defined as a score of 1 ("Very much improved") or 2 ("Much improved") on the Clinical Global Impression – Improvement (CGI-I) scale. The healthcare organization perspective (A) includes costs of the ERP or comparator interventions (i.e., the therapist-support time). The healthcare sector perspective (B) includes costs of the ERP or comparator interventions, healthcare visits, and medication/supplements. The societal perspective (C) includes costs of the ERP or comparator interventions, healthcare visits, medication/supplements, and other sector costs (e.g., productivity losses, child school absenteeism).

Abbreviations: ERP = therapist-supported internet-delivered exposure with response prevention for children and adolescents with Tourette syndrome or chronic tic disorder.

© 2024 Andrén P et al. *JAMA Network Open*.

eFigure 3. Cost-effectiveness acceptability curves with QALYs as the outcome for three costing perspectives

A. Healthcare organization perspective

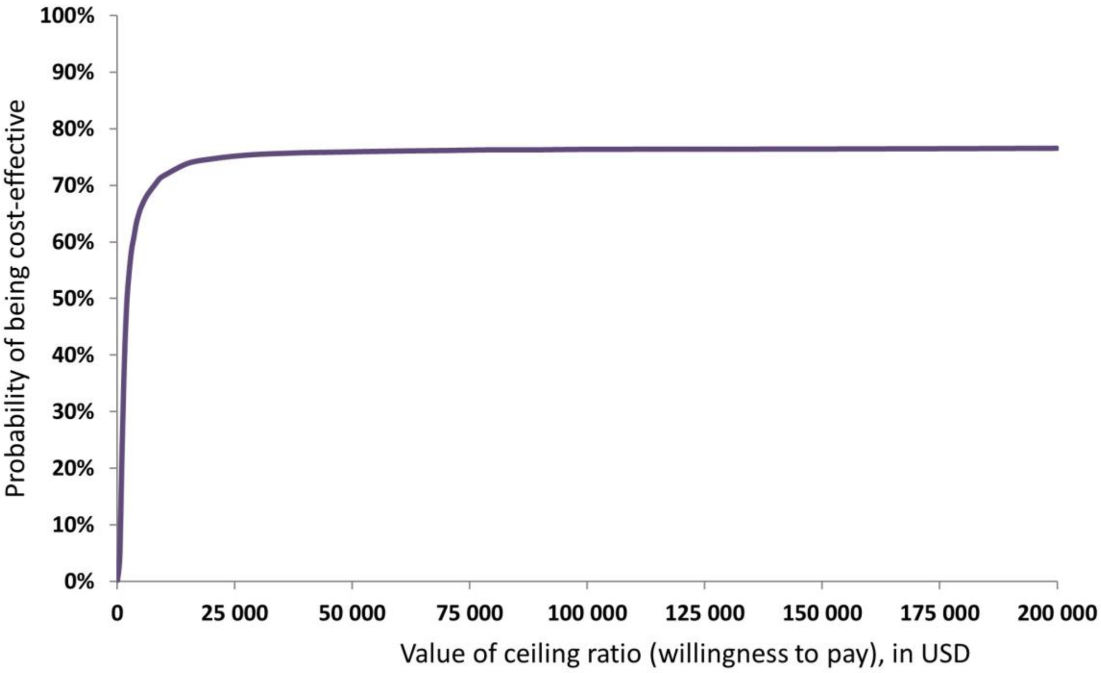

© 2024 Andrén P et al. *JAMA Network Open*.

B. Healthcare sector perspective

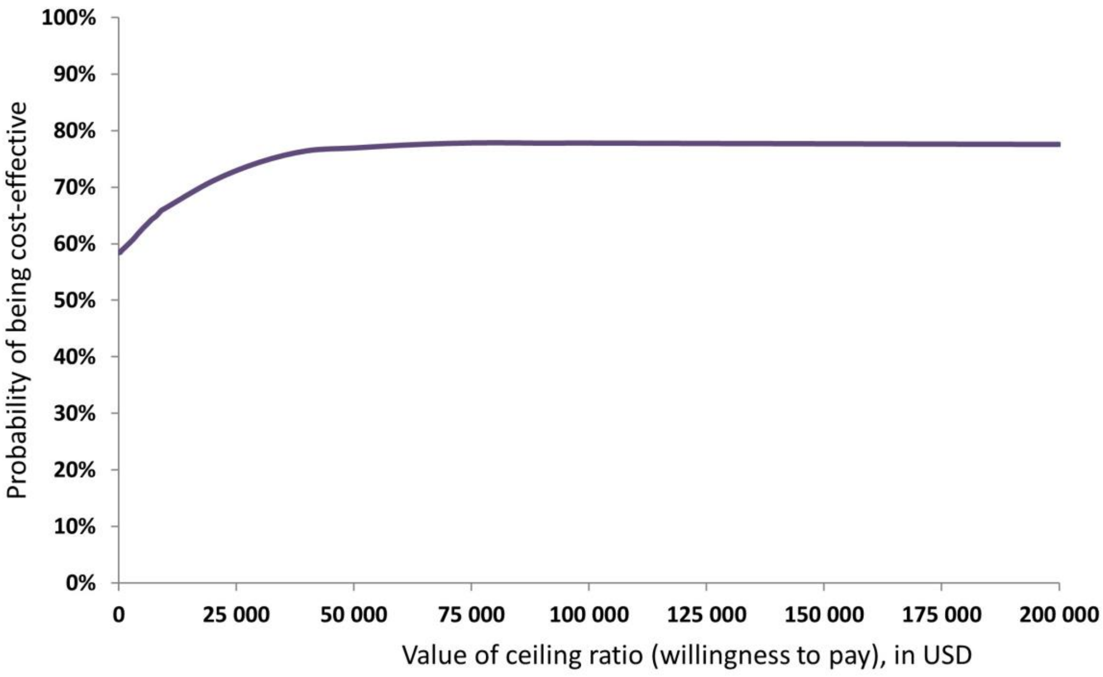

© 2024 Andrén P et al. *JAMA Network Open*.

C. Societal perspective

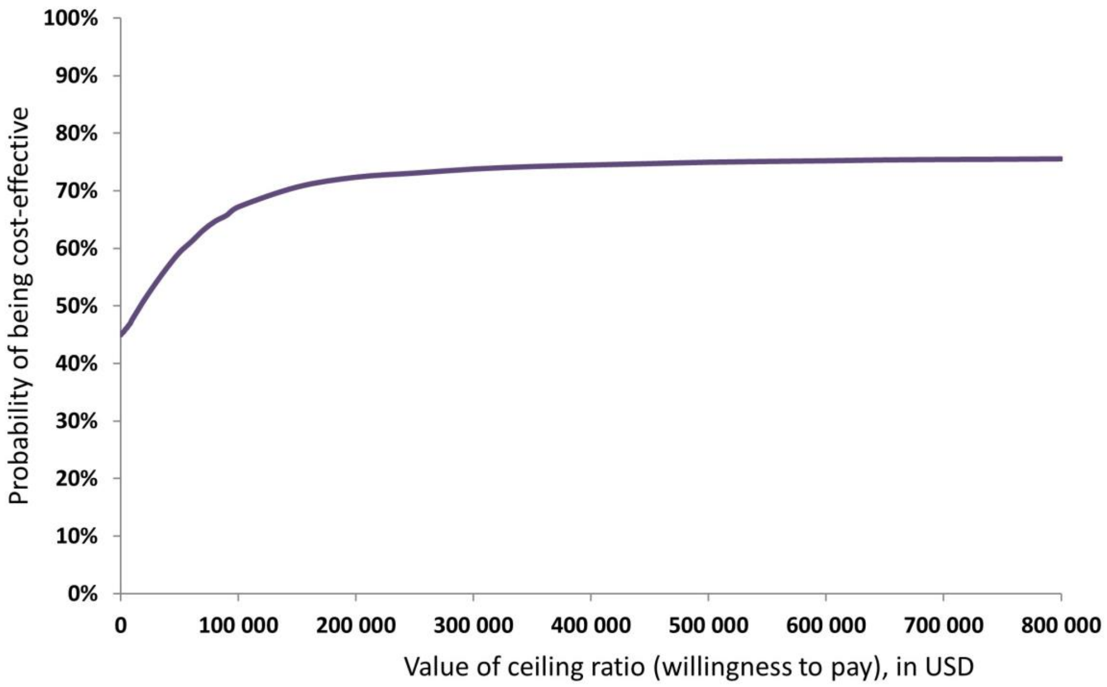

Note: The cost-effectiveness acceptability curves show the probability for the ERP intervention to be cost-effective at different willingness-to-pay levels (in USD), with QALYs as the outcome, for three costing perspectives. The healthcare organization perspective (A) includes costs of the ERP or comparator interventions (i.e., the therapist-support time). The healthcare sector perspective (B) includes costs of the ERP or comparator interventions, healthcare visits, and medication/supplements. The societal perspective (C) includes costs of the ERP or comparator interventions, healthcare visits, medication/supplements, and other sector costs (e.g., productivity losses, child school absenteeism).

Abbreviations: ERP = therapist-supported internet-delivered exposure with response prevention for children and adolescents with Tourette syndrome or chronic tic disorder; QALY = quality-adjusted life year.

© 2024 Andrén P et al. *JAMA Network Open*.

eReferences.

1. American Psychiatric Association. *Diagnostic and Statistical Manual of Mental Disorders: DSM-5*. 5th ed. American Psychiatric Publishing; 2013:xliv, 947 p.
2. Leckman JF, Riddle MA, Hardin MT, et al. The Yale Global Tic Severity Scale: initial testing of a clinician-rated scale of tic severity. *J Am Acad Child Adolesc Psychiatry*. Jul 1989;28(4):566-73. doi:10.1097/00004583-198907000-00015
3. Stevens K. Assessing the performance of a new generic measure of health-related quality of life for children and refining it for use in health state valuation. *Appl Health Econ Health Policy*. May 1 2011;9(3):157-69. doi:10.2165/11587350-000000000-00000
4. Chen G, Stevens K, Rowen D, Ratcliffe J. From KIDSCREEN-10 to CHU9D: creating a unique mapping algorithm for application in economic evaluation. *Health Qual Life Outcomes*. Aug 29 2014;12:134. doi:10.1186/s12955-014-0134-z
5. Matthews JN, Altman DG, Campbell MJ, Royston P. Analysis of serial measurements in medical research. *BMJ*. Jan 27 1990;300(6719):230-5. doi:10.1136/bmj.300.6719.230
6. Region Stockholm. Accessed 28 April, 2023. https://www.regionstockholm.se/sthlm-gotland
7. Swedish Association of Local Authorities and Regions. Accessed 28 April, 2023. https://skr.se/skr/halsasjukvard/ekonomiavgifter/kostnadperpatientkpp.1076.html
8. Apotea. Accessed 28 April, 2023. https://www.apotea.se/
9. Kigozi J, Jowett S, Lewis M, Barton P, Coast J. The Estimation and Inclusion of Presenteeism Costs in Applied Economic Evaluation: A Systematic Review. *Value Health*. Mar 2017;20(3):496-506. doi:10.1016/j.jval.2016.12.006
10. Statistics Sweden (Statistiska centralbyrån). Accessed 28 April, 2023. https://www.scb.se/hitta-statistik/sverige-i-siffror/lonesok/
11. Neumann PJ, Ganiats TG, Russell LB, Sanders GD, Siegel JE. *Cost-Effectiveness in Health and Medicine*. 2 ed. Oxford University Press; 2016:536.
12. Organisation for Economic Co-operation and Development (Purchasing Power Parities). Accessed 28 April, 2023. http://eppi.ioe.ac.uk/costconversion/
13. Schafer JL, Graham JW. Missing data: our view of the state of the art. *Psychol Methods*. Jun 2002;7(2):147-77.
14. Barber J, Thompson S. Multiple regression of cost data: use of generalised linear models. *J Health Serv Res Policy*. Oct 2004;9(4):197-204. doi:10.1258/1355819042250249
15. Manca A, Hawkins N, Sculpher MJ. Estimating mean QALYs in trial-based cost-effectiveness analysis: the importance of controlling for baseline utility. *Health Econ*. May 2005;14(5):487-96. doi:10.1002/hec.944
16. Drummond M, Sculpher M, Torrance G, O'Brien B, Stoddart G. *Methods for the economic evaluation of health care programmes*. New York, NY. 2005.
17. Fenwick E, Claxton K, Sculpher M. Representing uncertainty: the role of cost-effectiveness acceptability curves. *Health Econ*. Dec 2001;10(8):779-87. doi:10.1002/hec.635
18. Drummond MF, Sculpher MJ, Claxton K, Stoddart GL, Torrance GW. *Methods for the Economic Evaluation of Health Care Programmes*. Oxford: Oxford University Press; 2015.
19. Ekonomifakta. Accessed 28 April, 2023. https://www.ekonomifakta.se/fakta/skatter/skatt-pa-arbete/sociala-avgifter-over-tid

© 2024 Andrén P et al. *JAMA Network Open*.
