# Supplementary material for: Internet-Delivered Exposure and Response Prevention for Pediatric Tourette Syndrome: 12-Month Follow-Up of a Randomized Clinical Trial
Source: JAMA Netw Open. 2024 May 3;7(5):e248468. doi: 10.1001/jamanetworkopen.2024.8468 (PMC11069081; doi:10.1001/jamanetworkopen.2024.8468)
Supplement: Supplement 3. — Data Sharing Statement [file jamanetwopen-e248468-s003.pdf]

Data Sharing Statement

Andrén. Internet-Delivered Exposure and Response Prevention for Pediatric Tourette Syndrome. *JAMA Netw Open*. Published May 03, 2024.
doi:10.1001/jamanetworkopen.2024.8468

Data
Data available: No

Additional Information
Explanation for why data not available: The data are pseudonymised according to national (Swedish) and European Union legislation and cannot be anonymised and published in an open repository. Participants in the trial consent for their data to be shared with other international researchers for research purposes. The data can be made available upon reasonable request on a case-by-case basis according to the current legislation and ethical permits.
